# Analysis and interpretability of machine learning models to classify thyroid disease

**DOI:** 10.1371/journal.pone.0300670

**Published:** 2024-05-31

**Authors:** Sumya Akter, Hossen A. Mustafa

**Affiliations:** 1 Institute of Information and Communication Engineering, Bangladesh University of Engineering and Technology, Dhaka, Bangladesh; 2 Department of Computer Science and Engineering, Hajee Mohammad Danesh Science and Technology University, Dinajpur, Bangladesh; Abdul Wali Khan University Mardan, PAKISTAN

## Abstract

Thyroid disease classification plays a crucial role in early diagnosis and effective treatment of thyroid disorders. Machine learning (ML) techniques have demonstrated remarkable potential in this domain, offering accurate and efficient diagnostic tools. Most of the real-life datasets have imbalanced characteristics that hamper the overall performance of the classifiers. Existing data balancing techniques process the whole dataset at a time that sometimes causes overfitting and underfitting. However, the complexity of some ML models, often referred to as “black boxes,” raises concerns about their interpretability and clinical applicability. This paper presents a comprehensive study focused on the analysis and interpretability of various ML models for classifying thyroid diseases. In our work, we first applied a new data-balancing mechanism using a clustering technique and then analyzed the performance of different ML algorithms. To address the interpretability challenge, we explored techniques for model explanation and feature importance analysis using eXplainable Artificial Intelligence (XAI) tools globally as well as locally. Finally, the XAI results are validated with the domain experts. Experimental results have shown that our proposed mechanism is efficient in diagnosing thyroid disease and can explain the models effectively. The findings can contribute to bridging the gap between adopting advanced ML techniques and the clinical requirements of transparency and accountability in diagnostic decision-making.

## Introduction

The thyroid is a butterfly-shaped small gland situated in the middle of the throat. Thyroid hormone is produced through the secretion of the thyroid gland [1]. Some major body functions, including breathing, body weight control, heart rate, muscle strength, etc., are controlled by thyroid hormone. Triiodothyronine, also called T3, and Thyroxine, also called T4, are the two main hormones released by the thyroid gland. A little fluctuation from the desired amount of these hormones causes various types of thyroid disorders like hyperthyroidism, hypothyroidism, thyroid cancer, goiter, thyroiditis, Hashimoto’s thyroiditis, etc. [2].

According to data from the China Health Education Center, thyroid illness has become the second most common disease in the field of endocrinology. The number of thyroid patients is more than 200 million and is increasing at an alarming rate [3]. According to experts, 12% of people will experience a thyroid issue at any time in their lives. One in every eight women will develop a thyroid issue during their lifetimes, and women are around five to eight times more likely than men to suffer thyroid abnormalities [4]. American Thyroid Association claims that 20 million Americans suffer from thyroid illness in some way [5]. Additionally, an approximate calculation shows that 40% of the world’s population is in danger of an iodine nutrient deficit, which is crucial for producing hormones of thyroid [3]. These numbers indicate that thyroid disease should not be handled lightly. Modern technologies should be used to advance medical methods for diagnosing and treating thyroid diseases.

The most difficult task is dealing with one’s life, which physicians often face. To give proper treatment, it is mandatory to diagnose any disease accurately and quickly. To make our everyday tasks easy, Artificial Intelligence (AI) came into existence. Many fields, including healthcare, are now encompassed by Machine Learning (ML) models. ML models are used to detect various diseases in the early stage, which helps physicians make decisions efficiently. Among various types of diseases, in this work, we focus on thyroid disease as it appears to have received comparatively less attention from the medical community as a whole [6]. In the healthcare industry, classification statistics have shown that only 15% of research is based on thyroid disease, whereas breast cancer and heart disease research are about 40% and 20% respectively [1].

For classification using ML, dataset balancing is one of the powerful preprocessing techniques in ML. There are several data balancing techniques to balance the imbalanced dataset to predict the target value more accurately. This concept is applied by many researchers in various domains such as healthcare, security, etc. Among numerous research on the healthcare domain, to diagnose hepatitis [7], oversampling and undersampling methods were used to compare the performance of the model. Finally, they found that the oversampling method performed better. In [8], a new approach was implemented to balance the imbalanced dataset. They proposed a cluster-based oversampling technique combining the Synthetic Minority Oversampling Technique (SMOTE) and the k-means algorithm. To overcome the drawbacks of SMOTE, a balancing technique was introduced combining an oversampling method called SMOTE and an undersampling method called Edited Nearest Neighbour (ENN) [9]. This hybrid method, SMOTE-ENN, was used to predict heart disease by Jian Yang et al. [10], which increased the classifiers’ performance, proving the importance of data balancing in healthcare. The authors [11] used SMOTE, NearMiss, and SMOTETomek as data balancing techniques and applied ML and ensemble ML models to check the priority of the balancing techniques on heart disease. To predict stroke using ML methods among the older Chinese in a high imbalance dataset, authors used SMOTE in the preprocessing phase and got a significant result on the performance of the classifiers. It helped the classifiers to show a stable result and improved the accuracy of the classifiers at a reasonable rate [12]. In 2022 [13], authors made an analysis showing that k-means SMOTE-ENN performs better than other balancing techniques. Islam et al. [14] proposed a two-stage data balancing framework where they combined Adaptive Synthetic Sampling (ADASYN), Support Vector Machine (SVM)-SMOTE, and SMOTE+ENN characteristics and achieved good results.

Machine learning is a field that can improve our lives if used in the healthcare domain. Researchers have been working in this field to make a convenient and efficient healthcare system. Recently, machine learning as well as deep learning models have been applied to detect thyroid disease accurately [15]. The authors of [4] conducted an analysis of thyroid disease using six machine learning algorithms: Support Vector Machine (SVM), AdaBoost (AdB), Decision Tree (DT), Gradient Boosting (GB), K Nearest Neighbour (KNN), and Random Forest (RF) with 5-fold cross-validation. They used SMOTE for balancing and three feature selection techniques: Boruta, Recursive Feature Elimination (RFE), and Least Absolute Shrinkage and Selection Operator (LASSO). Among all the combinations, RF+LASSO showed the best accuracy score. In [5], R. Chaganti et al. presented five machine learning models and three deep learning models in addition to four feature selection strategies. They used Forward Feature Selection (FFS), Backward Feature Elimination (BFE), BiDirectional Feature Elimination (BiDFE), and Machine Learning-based Feature Selection using extra tree classifiers (MLFS) to select features along with RF, SVM, AdB, GB as ML algorithms and Convolutional Neural Network (CNN), Long Short Term Memory (LSTM) and CNN-LSTM as Deep Learning (DL) algorithm. In this study [16], some preprocessing techniques like data augmentation and import Alexnet were used in the training session. They compared four classifier performances: Artificial Neural Network (ANN), KNN, DT, and RF, where RF had given maximum accuracy. An extensive analysis was presented in [17], where the authors applied SMOTE for data balancing and Principle Component Analysis (PCA) for feature selection with ten machine learning classifiers. After analysis of all of the classifiers i.e., ANN, CatBoost, eXtreme Gradient Boosting (XGBoost), RF, Light Gradient Boosting Machine (LightGBM), DT, SVM, KNN, Extra Tree (ET), and Gaussian Naive Bayes (GNB); ANN showed the highest accuracy in detecting thyroid disease. Jha et al. [18] employed their work in two stages. In the first stage, they worked with neural networks (NN) and KNN with different values of K = 3,5,7 using three feature selection techniques: PCA, Singular Value Decomposition (SVD), and DT. In the second stage, they applied a Deep Neural Network (DNN) after using data augmentation. DT+NN gave the best result in the first stage, and DNN exceeded the first-stage result. A simple analysis was shown in [19] with two small datasets where they compared the accuracy with three ML models: Logistic Regression (LR), DT, and KNN. In [20], the best features were selected using the XGBoost function, and four classifier performances were compared: XGBoost, DT, LR, and KNN, where XGBoost gave the highest accuracy. In [21], at first, authors scaled the features and different feature selection methods, including Pearson’s Correlation (PC), Symmetrical Uncertainty (SU), one-R classifier, and Relief Attribute Evaluation (RAE) were used with Multiple Multi-Layer Perceptron (MMLP) classifiers. They found that MMLP+Pearson’s correlation produced the best accuracy.

Furthermore, machine learning models are not only employed for classifying various diseases but also for predicting numerous biological disorders. A multitude of machine learning-based computational models has been introduced for protein sample prediction [22]. Ali et al. [23] introduced the AFP-CMBPred predictor for antifreeze protein identification and performed protein sample classification using SVM and RF-based learning models. In the field of biomedical engineering, several other predictors used ML and XAI methods [24], e.g., DP-BINDER [25] for prediction of DNA-binding proteins, iAFPs-EnC-GA [26] for antifungal peptides prediction, iRNA-PseTNC [27] for identification of RNA 5-methylcytosine sites, and cACP-DeepGram [22] for classification of anticancer peptides.

However, these models are not explainable because of their black-box nature. To remove this barrier, explainable artificial intelligence (XAI) has been introduced recently, where predictions are explained by AI systems [28]. XAI has gained high popularity as it can explain ML diagnosis of any disease locally and globally, which is understandable by everyone and can ensure the trust of healthcare experts in AI. Although more and more people are turning to explainability methods to make sense of the black box in which machine learning models operate, a few works have been done on the thyroid dataset. In 2022, the explainability approach, SHapley Additive exPlanations (SHAP), was used for both global and local interpretability for the thyroid disease dataset based on only one applied model, which is logistic regression by SK. Arjaria et al. [29], but no preprocessing techniques were used. Aljameel et al. [30] applied an Explainable Artificial Neural Network (EANN) model for the dataset of thyroid cancer and used SMOTE-ENN for data balancing. In this study, they used SHAP only for global interpretability. In 2023 [31], authors recently applied seven machine learning algorithms with some preprocessing steps to predict hypothyroidism and hyperthyroidism. They also used SHAP and Locally Interpretable Model Agnostic Explanation (LIME) to interpret the model. Besides this, mapping between the domain expert’s knowledge and the XAI explainability should be investigated to cross-check the performance of the existing XAI tools and the effectiveness of explainability in the healthcare domain.

Most of the related works focused on designing an ML-based methodology to predict thyroid disease. The focus is on the overall accuracy of the model, but there is limited analysis of how a model shows better accuracy compared to others. In other cases, the authors only applied the XAI tools for model interpretability but didn’t try to map or cross-check the output with real-world explainability with domain experts. We have tried to address these limitations in this paper.

In this paper, we have addressed the above issues and made the following contributions:

- Our research contributed innovative cluster-based data balancing techniques designed to address the limitations of existing methods. These techniques aim to prevent overfitting and remove noise from data to enhance model performance and robustness.
- We proposed an ML-based methodology featuring an extensive preprocessing pipeline, contributing to more effective and accurate thyroid disease prediction. This novel approach has the potential to improve healthcare outcomes and patient diagnostics.
- Our research contributed to the field of model explainability by employing eXplainable Artificial Intelligence (XAI) techniques. We demonstrated how these techniques can provide both global and local explainability for features in the context of thyroid disease prediction, shedding light on the “black-box” nature of machine learning models.
- We contributed to understanding the alignment between XAI tools and expert opinions through a comprehensive survey. This research identified and analyzed gaps in current XAI tools to expert perspectives, paving the way for improved model interpretability and decision support in the healthcare domain.

The rest of the paper is organized as follows: The proposed methodology with the experimental setup is presented in the section Materials and Methods. Section Results and Discussion contains the experimental results and discussion about the overall outcome of our proposed scheme. Finally, the Conclusion section concludes the paper with future work.

## Materials and methods

**Fig 1:**
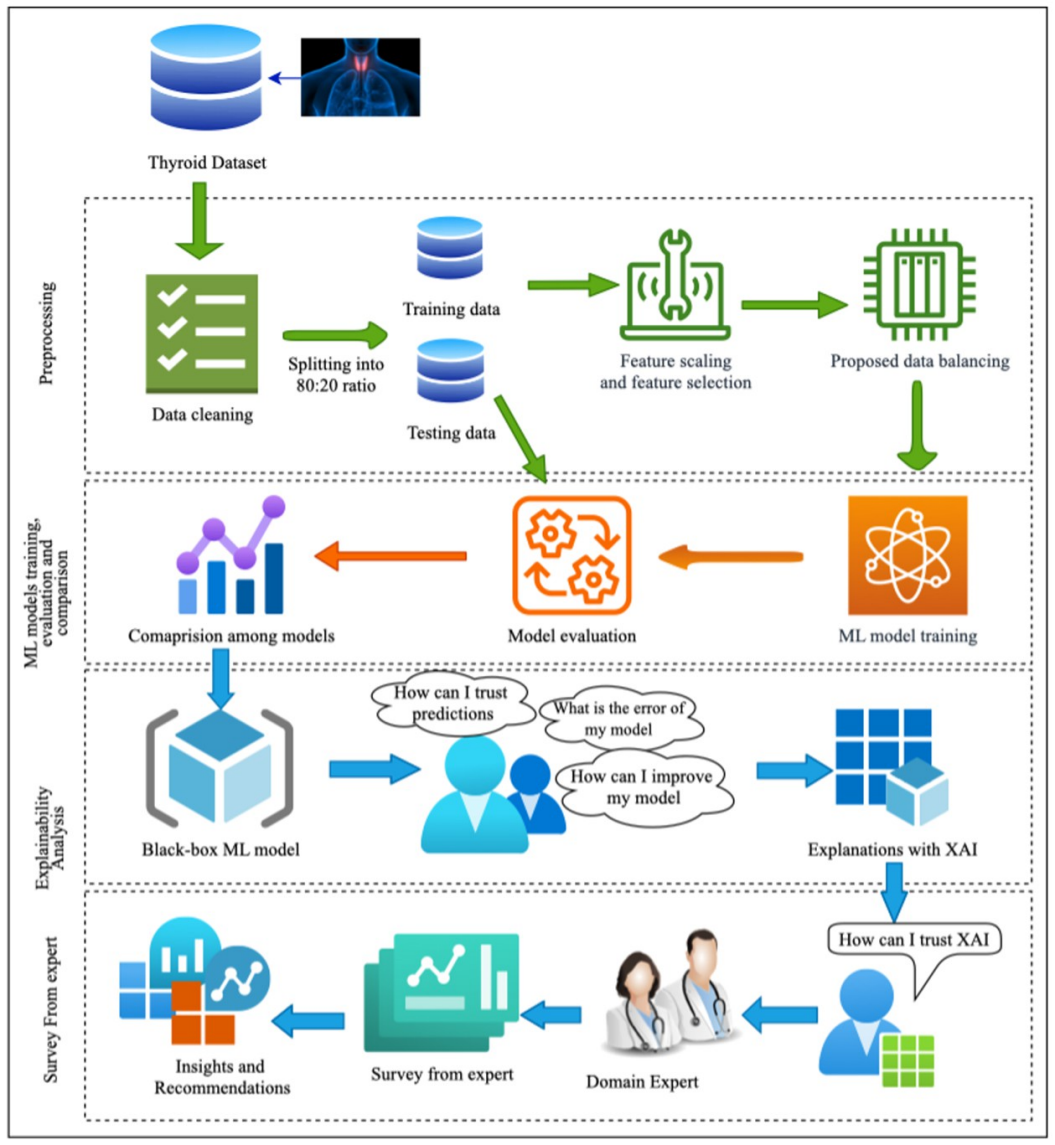
Proposed scheme for thyroid disease prediction and explanation.

This section describes the proposed scheme in detail. The block diagram of our scheme is shown in Fig 1. The scheme is divided into four major segments: (i) Data preprocessing, (ii) Model building using ML classifiers, (iii) Model explainability, and (iv) Survey from domain experts.

### Data preprocessing

The thyroid disease dataset used in our proposed scheme is originally from the University of California, Irvine (UCI) machine learning repository. Then, it was modified by Goldstein Markus et al. in 2015 [32]. This dataset has 16 categorical attributes, 5 numerical attributes, and 1 target attribute; 22 attributes in total. This dataset contains 6916 instances: 6666 in class 0 and 250 in class 1. The features of our dataset are explained in Table 1.

**Table 1:** Features of the dataset.

Serial No.	Feature Name	Feature Type	Description
1	Age	float	Age of the patient
2	Sex	float	Defines sex (Male or Female)
3	on_thyroxine	float	Whether patient is on thyroxine
4	query_on_thyroxine	float	Whether patient is on thyroxine
5	on_antithyroid_medication	float	Whether patient takes antithyroid medication
6	sick	float	Whether patient is sick
7	Pregnant	float	Whether patient is pregnant
8	thyroid_surgery	float	Whether patient have any surgery
9	I131_treatment	float	Whether patient is receiving I131 treatment
10	query_hypothyroid	float	Whether patient thinks they have hypothyroidism
11	query_hyperthyroid	float	Whether patient thinks they have hyperthyroidism
12	lithium	float	Whether patient has lithium
13	goitre	float	If patient has a goiter
14	tumor	float	If patient has a tumor
15	Unnamed:22	float	NULL values
16	psych	float	Whether patient psych
17	TSH	float	TSH level in the blood
18	T3_measured	float	T3 level in the blood
19	TT4_measured	float	TT4 level in the blood
20	T4U_measured	float	T4U level in the blood
21	FTI_measured	float	FTI level in the blood
22	Outlier_label	Object	Thyroid classification (Yes or No)

The preparation of the dataset is necessary for the dataset analysis. Based on the characteristics of the dataset, different preprocessing techniques are applied to convert the datasets into more ML-centric ones to fit the ML classifiers accurately. At first, we cleaned the data by handling outliers and inconsistencies. Then, we normalized or standardized numerical features to bring them to a common scale and encoded categorical variables. We used the random forest algorithm to perform feature analysis and identified the most relevant features that contributed to thyroid disease prediction. As our dataset is highly imbalanced, we proposed two data-balancing techniques to balance the dataset.

#### Proposed data balancing techniques

**Fig 2:**
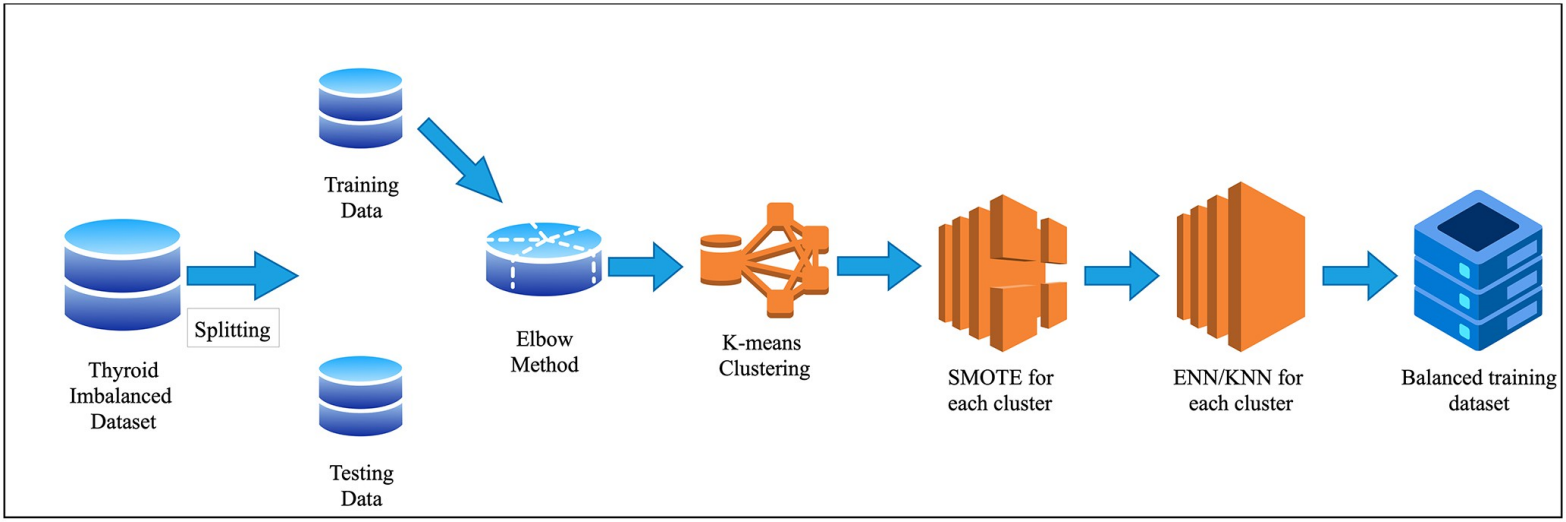
Proposed data balancing technique.

To balance the imbalanced dataset, we proposed two data-balancing techniques. The top-down view of the proposed methodology is shown in Fig 2.

Our proposed data balancing techniques used a hybrid data balancing process, combining an oversampling and an under-sampling method with a clustering technique to balance the dataset. We divided the dataset into several clusters based on the data characteristics determined by K-means clustering [33]. To find the optimal number of clusters, we used the elbow method [34]. Before applying k-means clustering, we split the dataset into training and testing with an 80:20 ratio.

Subsequently, we initiated the K-means clustering process by specifying a range of possible values for k. For each k in this range, we computed the sum of squared distances (SSD) from every data point to its designated cluster centroid. We then constructed a graphical plot with k values along the x-axis and their corresponding SSD values along the y-axis. While analyzing the plot, we noticed the relationship between k and the decrease in SSD. Ultimately, we identified the “elbow” point on the plot, signifying where the SSD starts to exhibit a noticeable slowing down. This “elbow” point signified the optimal number of clusters.

After that, we divided the training data into five clusters, which are determined by the elbow method. In each cluster, we applied an oversampling technique SMOTE. After applying SMOTE, some noises were added. In our first technique, we removed the noise by applying the ENN undersampling technique; we referred to this as SMOTE+ENN. In the second technique, we used KNN as the undersampling technique, and we referred to this as SMOTE+KNN. After applying SMOTE+ENN or SMOTE+KNN on training data in each cluster, we merged all the cluster data and created the final balanced training dataset. In this process, we preserved our testing data the same as the original data. Finally, ML models are trained by training data, and we tested the ML models using testing data. The algorithm of our proposed data balancing technique, SMOTE+ENN, is shown in Algorithm 1.

**Algorithm 1** K-Means SMOTE+ENN Data Balancing

1: **Input:** *X* (dataset), *k*_max_ (maximum number of clusters), *k*_neighbors1_ (number of SMOTE neighbors), *k*_neighbors2_ (number of ENN neighbors)

2: **Output:** *X*_balanced_ (balanced dataset)

3: *distortions* ← ∅

4: **for** *k* = 1 to *k*_max_

5:  Cluster the data points in *X* into *k* clusters using K-Means algorithm

6:  *distortions*[*k*] ← sum of squared distances (SSD) of data points to their nearest cluster centroid

7: **Endfor**

8: Plot a graph with *k* values on the x-axis and the corresponding SSD on the y-axis

9: Identify the elbow point with the sharpest decrease in SSD as the optimal number of clusters.

10: Choose the optimal *k* using the elbow method that corresponds to the elbow point.

11: Cluster the data points in *X* into the optimal *k* clusters using K-Means algorithm to obtain the final clustering results

12: **for** *i* = 1 to *k*

13:  *X*_*i*_← data points in cluster *i*

14:  Compute the centroid *C*_*i*_ of cluster *X*_*i*_

15: **Endfor**

16: *X*_smote_ ← ∅

17: **for** *i* = 1 to *k*

18:  **for** datapoint *x* in *X*_*i*_

19:   Find the *k*_neighbors1_ nearest neighbors of *x* in *X*_*i*_

20:    **for** each nearest neighbor *n*

21:     Generate a synthetic example *s* by applying SMOTE between *x* and *n*

22:     *X*_smote_ ← *X*_smote_ ∪ {*s*}

23:    **Endfor**

24:   **Endfor**

25: **Endfor**

26: *X*_smotebalanced_ ← *X* ∪ *X*_smote_

27: Use ENN (Edited Nearest Neighbors) to remove noisy examples from *X*_smotebalanced_

28: Find the nearest *k*_neighbors2_ neighbors for each data point in *X*_smotebalanced_

29: Remove data points whose class label differs from the majority class of its *k*_neighbors2_ neighbors

30: **return** *X*_balanced_

#### Existing data balancing techniques

*SMOTE*. Synthetic Minority Over-sampling Technique is an oversampling data balancing technique that is commonly used in machine learning to address the problem of imbalanced classes [35]. It works by creating synthetic examples of the minority class by interpolating between existing examples.

*NearMiss*. NearMiss is an undersampling technique used to balance imbalanced datasets [36]. This technique selects the examples from the majority class that are closest to the examples of the minority class and keeps only a subset of them. The subset is determined based on the parameter used for this technique.

*SMOTEENN*. SMOTE is an oversampling technique that creates some noise or outliers in the dataset. To mitigate this problem, Batista et al. [9] proposed a hybrid approach combining SMOTE with Edited Nearest Neighbour(ENN), which is an undersampling technique. SMOTEENN combines these two techniques to create a hybrid approach. By default, ENN considers three nearest neighbors for each data point.

*SMOTEKNN*. This is also a hybrid procedure to handle noisy imbalance data where SMOTE combines with K Nearest Neighbour(KNN). SMOTEKNN performs upsampling and downsampling at the same time. By default, KNN is taken from the five nearest neighbors for each data point.

### Model building using ML classifiers

We use the following nine (9) ML models with 5-fold cross-validation to build our proposed scheme for analysis.

#### Adaptive Boosting (AdaBoost)

Adaptive Boosting (AdaBoost) is a popular ensemble learning algorithm used for binary classification tasks [37]. It works by combining multiple weak learners, typically decision trees, to create a strong learner that can make accurate predictions on new data [38].

#### Decision Tree (DT)

A Decision Tree (DT) is a popular ML algorithm used for both classification and regression problems. It works by recursively partitioning the input data into subsets based on the value of one or more input features and creating a tree-like model of decisions that lead to a prediction or outcome [39].

#### Extreme Gradient Boosting (XGB)

Extreme Gradient Boosting (XGB) is a gradient-boosting algorithm designed for tree-based models. In the case of XGB, the optimal model is selected by taking into account more precise approximations. The loss functions are computed to reduce loss, and enhanced regularization is used to lessen overfitting and increase model performance [37].

#### Extra Tree Classifier (ETC)

The Extra Tree Classifier(ETC) is an ensemble learning method for classification tasks. It is an extension of the Random Forest algorithm and belongs to the family of decision tree-based ensemble models. The ETC aims to reduce overfitting and improve the generalization performance compared to traditional decision trees. By introducing more randomness, ETC can handle noisy data more effectively and may be computationally more efficient compared to Random Forest [40].

#### K Nearest Neighbour (KNN)

K-Nearest Neighbor (KNN) works by finding the k closest data points in the training set to a new input instance using similarity metrics such as Euclidean distance function [41]. KNN has applications in both classification and regression.

#### Logistic Regression (LR)

Logistic Regression (LR) is a statistical method used to analyze the relationship between a binary dependent variable and one or more independent variables. It is commonly used for binary classification problems, where the dependent variable can take only two values, i.e., zero or one [42].

#### Multilayer Perceptron (MLP)

Multilayer Perceptron (MLP) is the fundamental architecture of deep learning, sometimes referred to as the feed-forward artificial neural network. An input layer, one or more hidden layers, and an output layer make up a standard MLP, which is a fully connected network [37].

#### Random Forest (RF)

Random Forest (RF) is a popular ML algorithm that belongs to the family of ensemble methods. It works by constructing multiple decision trees and combining their outputs to make a final prediction [43].

#### Support Vector Machine (SVM)

Support Vector Machine (SVM) is a popular ML algorithm that can be used for both classification and regression tasks. It works by constructing a hyperplane or a set of hyperplanes that separates the input data into different classes or predicts a continuous output variable. It works well in high-dimensional spaces and can act differently depending on certain mathematical functions called kernels. The typical SVM classifier kernel functions include linear, polynomial, radial basis function (RBF), sigmoid, etc. [37]

### Model explainability

To overcome the difficulty of explaining ML models due to their black-box nature, XAI has been introduced and gained popularity [44]. In our scheme, we tried to explain the model to identify the global and local interpretability to make it understandable for everyone. We generated explanations for individual predictions to understand the factors influencing the model’s decision-making process. After that, we visualized feature importance scores and contribution values to identify which features contribute most to a particular prediction. We used two most popular XAI tools SHAP and Shapash for implementation.

#### SHAP

SHAP (SHapley Additive exPlanations) is an Explainable AI (XAI) technique used to explain the output of machine learning models [45]. It is based on the concept of Shapley values from cooperative game theory and provides a way to decompose the output of a machine learning model into contributions from each input feature. This enables a better understanding of how the model makes predictions and can be used for feature selection, model debugging, and building trust with stakeholders [46].

#### Shapash

Shapash is an open-source Explainable AI (XAI) tool that provides interactive visualizations to explain the output of machine learning models [47]. It is designed to be user-friendly and does not require any coding or machine-learning expertise. The tool allows users to explore the data and model predictions and generate explanations for specific predictions. The explanations include visualizations of the contributions of each input feature to the prediction and can be used to understand the reasons behind the model’s output and to identify areas where the model may be improved.

### Survey from domain experts

We surveyed the domain experts (Doctors and Medical senior students) to find the weight and rank of the features. Firstly, we conducted face-to-face interviews with five doctors and asked them to select variables from the dataset list. According to their opinion, we counted the frequency and ranked the features. Then, we conducted another survey among final year medical students with a questionnaire and asked them some questions and put the value corresponding to each feature from 1 to 5, where 5 means the features are more important and 1 indicates less importance.

By following this methodology, we can build a thyroid disease prediction model that provides accurate predictions and offers insights and recommendations into the factors driving those predictions, making it more transparent and trustworthy for clinical decision-making.

#### Ethical statement

All participants gave written informed consent before the survey. The consent form is added as an additional document.

## Results and discussion

### Performance measure metrics

To find the best-performing ML algorithm, we need to measure the performance of the classifiers using some performance measure metrics.

#### Confusion matrix

Confusion matrix is one of the performance measure metrics, which measures the performance of the classification algorithm as represented in Table 2.

**Table 2:** Confusion matrix.

		Predicted class	
		0	1
Actual class	0	True Negative (TN)	False Positive (FP)
	1	False Negative (FN)	True Positive (TP)

#### Accuracy

Accuracy is a common performance measure metric used in classification tasks. It represents the proportion of correct predictions made by a model.

$$Accuracy=\frac{\left(TP+TN\right)}{\left(TP+TN+FP+FN\right)}$$

#### Precision

It measures the accuracy of positive predictions made by the model, specifically the ratio of true positive predictions to the total number of positive predictions.

$$Precison=\frac{TP}{TP+FP}$$

#### Recall

It measures the ability of a model to correctly identify all relevant instances of a specific class, typically the ratio of true positive predictions to the total number of actual positive instances.

$$Recall=\frac{TP}{TP+FN}$$

#### F1-score

It is interpreted as a harmonic mean of the precision and recall. The formula for calculating F1-score is given in the following Equation.

$$F1-Score=\frac{2*Precision*Recall}{Precision+Recall}$$

#### Precision-recall curve

The precision-recall curve is created by plotting precision against recall for different classification thresholds. Each point on the curve represents a different threshold used to classify instances as positive or negative. The curve helps to visualize the trade-off between precision and recall. A model with high precision and high recall will have a curve that hugs the upper-right corner of the plot.

#### Area Under the Curve (AUC)

The AUC measures how efficiently the model differentiates between negative and positive classes. Its value varies from 0 to 1, with a higher AUC value indicating better performance.

#### Receiver Operating Characteristic Curve (ROC Curve)

The ROC curve gives a graphical representation of the classifier’s performance at various thresholds. ROC curve helps to visualize, organize, and choose classifiers according to their performance.

### Experimental setup

In our scheme, we used two different data balancing techniques and compared them with four existing data balancing techniques. The number of instances is not the same among different data balancing techniques. In Table 3, we have shown the state of the data count before and after data balancing.

**Table 3:** Number of instances before and after data balancing.

State	Thyroid Dataset	
Class	Class 0	Class 1
Original dataset	6666	250
Size of Training data (80 percent of original data)	5340	192
Size of Testing data (20 percent of original data)	1326	58
After Applying SMOTE in training data	5340	5340
After applying NearMiss in training data	192	192
SMOTE+ENN in training data	4525	4994
SMOTE+KNN in training data	4774	5272
K-means clustering+SMOTE+ENN(Proposed)	5340	5025
K-means clustering+SMOTE+KNN(Proposed)	5010	5265

This table shows that the dataset is imbalanced, and after applying the balancing techniques, it is balanced; sometimes, the instances increase and sometimes decrease. We used the balanced training dataset separately with different ML classifiers and checked the results using the classification report.

The associated hyperparameters in the ML classifiers are determined with grid search cross-validation (CV). For all the ML algorithms, we used 5-fold cross-validation to avoid overfitting. All the hyperparameters and their respective values are provided in Table 4.

**Table 4:** Hyperparameters tuning of the classifiers using gridsearchCV.

ML Classifier	Parameter and Value
AdaBoost	*n_estimators* = 50, *learning_rate* = 0.01, *random state* = 42
DT	*criterion*=‘gini’, *splitter*=‘best’, *random state*=42
ETC	*n_estimators* = 100, *random state* = 42,
KNN	*n_neighbpurs* = 5, *leaf_size* = 30, *p* = 2, *metric* = ‘minkowski’
LR	*penalty* = ‘l2’, *C* = 1.0, *solver* = ‘liblinear’, *max_iter* = 100, *multi_class* = ‘auto’, *verbose* = 0, *random state* = 42
MLP	*hidden_layer_sizes* = 100, *activation* = ‘relu’, *solver* = ‘adam’, *alpha* = 0.0001, *batch_size* = ‘auto’, *random state* = 42
RF	*n_estimators* = 100, *random state* = 42
SVM	*C* = 1.0, *gamma* = ‘scale’, *kernel* = ‘rbf’, *verbose*=False”, *probability* = ‘True’, *random state* = 42
XGB	*eval_metric* = ‘mlogloss’, *learning_rate* = 0.1, *n_estimators* = 100, *random state* = 42

### Performance of our proposed scheme

In this section, we discussed the performance of our proposed scheme, where we applied our proposed balancing techniques: K-means+SMOTE+ENN and K-means+SMOTE+KNN. As discussed, we analyzed nine ML models in our experiments. Tables 5 and 6 showed the performance of our scheme for the 9 ML models. All the performance measures presented in the following are for the test data applied after the models are trained.

**Table 5:** Performance measure of our scheme using K-means+SMOTE+ENN.

ML Models	Accuracy	Precision	Recall	F1 score	AUC
AdaBoost	0.9833	0.9866	0.9833	0.9843	0.9981
DT	0.9833	0.9830	0.9833	0.9831	0.884
ETC	0.9732	0.9703	0.9732	0.9707	0.9617
KNN	0.9212	0.9465	0.9212	0.9321	0.7218
LR	0.8988	0.9462	0.8988	0.9186	0.8018
MLP	0.9588	0.9605	0.9588	0.9596	0.8487
RF	0.9833	0.9835	0.9833	0.9834	0.9945
SVM	0.9638	0.9615	0.9638	0.9625	0.8480
**XGB**	0.9913	0.9919	0.9913	0.9915	0.9981

**Table 6:** Performance measure of our scheme using K-means+SMOTE+KNN.

ML Models	Accuracy	Precision	Recall	F1 score	AUC
AdaBoost	0.9826	0.9872	0.9826	0.9839	0.9977
DT	0.9862	0.9866	0.9862	0.9864	0.9268
ETC	0.9725	0.9705	0.9725	0.9713	0.9547
KNN	0.9031	0.9449	0.9031	0.9209	0.7369
LR	0.8707	0.9436	0.8706	0.9010	0.7977
MLP	0.9291	0.9541	0.9291	0.9394	0.8526
RF	0.9841	0.9850	0.9841	0.9844	0.9931
SVM	0.8988	0.9507	0.8988	0.9198	0.8448
**XGB**	0.9899	0.9911	0.9898	0.9903	0.9970

As we can see in Table 5, the XGB classifier got the highest score (over 99%) in all performance metrics among all the classification algorithms. The performance of AdaBoost, DT, and RF are similar, with 98.33% accuracy. On the other hand, the performance of LR is the worst, with 89.88% accuracy.

As we can see in Table 6, after applying K-means+SMOTE+KNN technique for data balancing, the performances of most of the models are a bit lower compared to K-means+SMOTE+ENN. Again, XGB has given the best performance with more than 98.99% accuracy, 99.11% precision, and 99.03% F1-score. The accuracy of AdaBoost, DT, and RF are similar, with more than 98% accuracy. In contrast, LR and SVM have the lowest performance in this case, with 87.07% and 89.88% accuracy, respectively.

In our experiment, we employed a range of ML methods: neural network models, tree-based models, and statistical models. We observed that the performance of tree-based classifiers such as DT, RF, XGB, Ada, ETC are higher than others. Tree-based ML algorithms beat other methods in these experiments primarily because they didn’t need pre-processing characteristics like standardization or normalization. The second factor was the need for bagging and boosting in ML models based on trees. Among tree-based models, the XGB classifier performed the best in this experiment, and it used boosting to improve the model’s performance. Unlike bagging, boosting generated models one by one. Since each base model learned from the flaws of the previous model, boosting increased accuracy. XGBoost performed admirably when overfitting occurs, as in our case, where the train and test data were not balanced [17].

**Fig 3:**
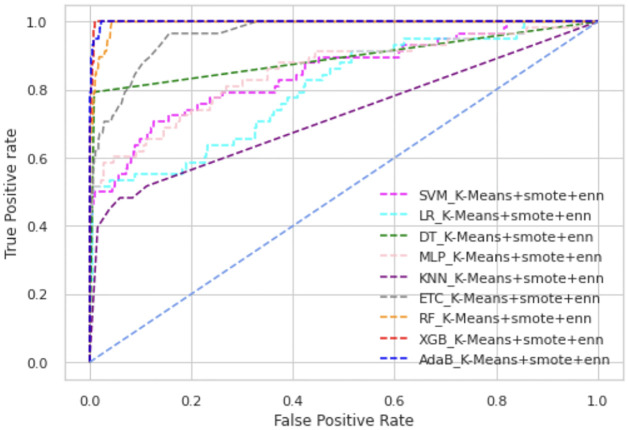
ROC curve of K-means+SMOTE+ENN.

**Fig 4:**
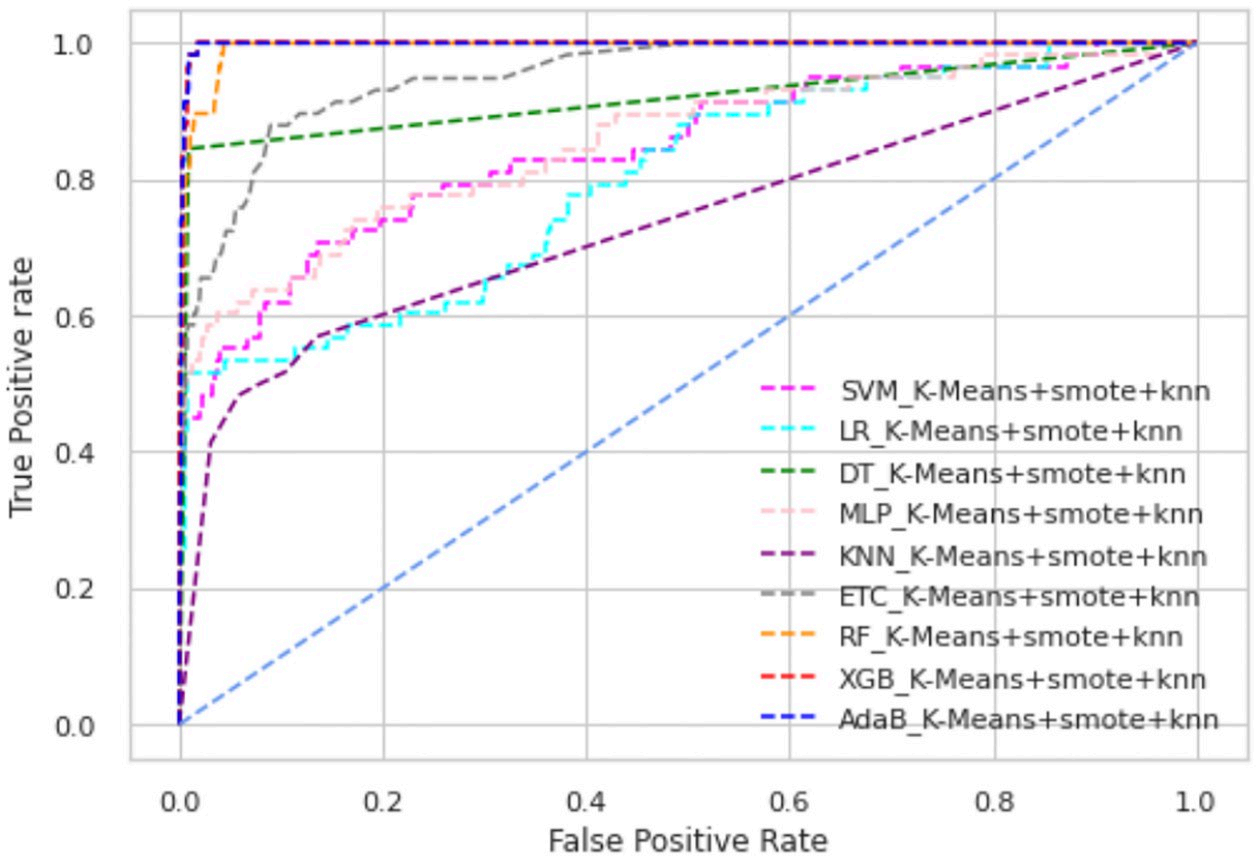
ROC curve of K-means+SMOTE+KNN.

In Figs 3 and 4, we have presented the ROC curve, another performance measure metric to show the performance of all the ML models of our proposed scheme.

**Fig 5:**
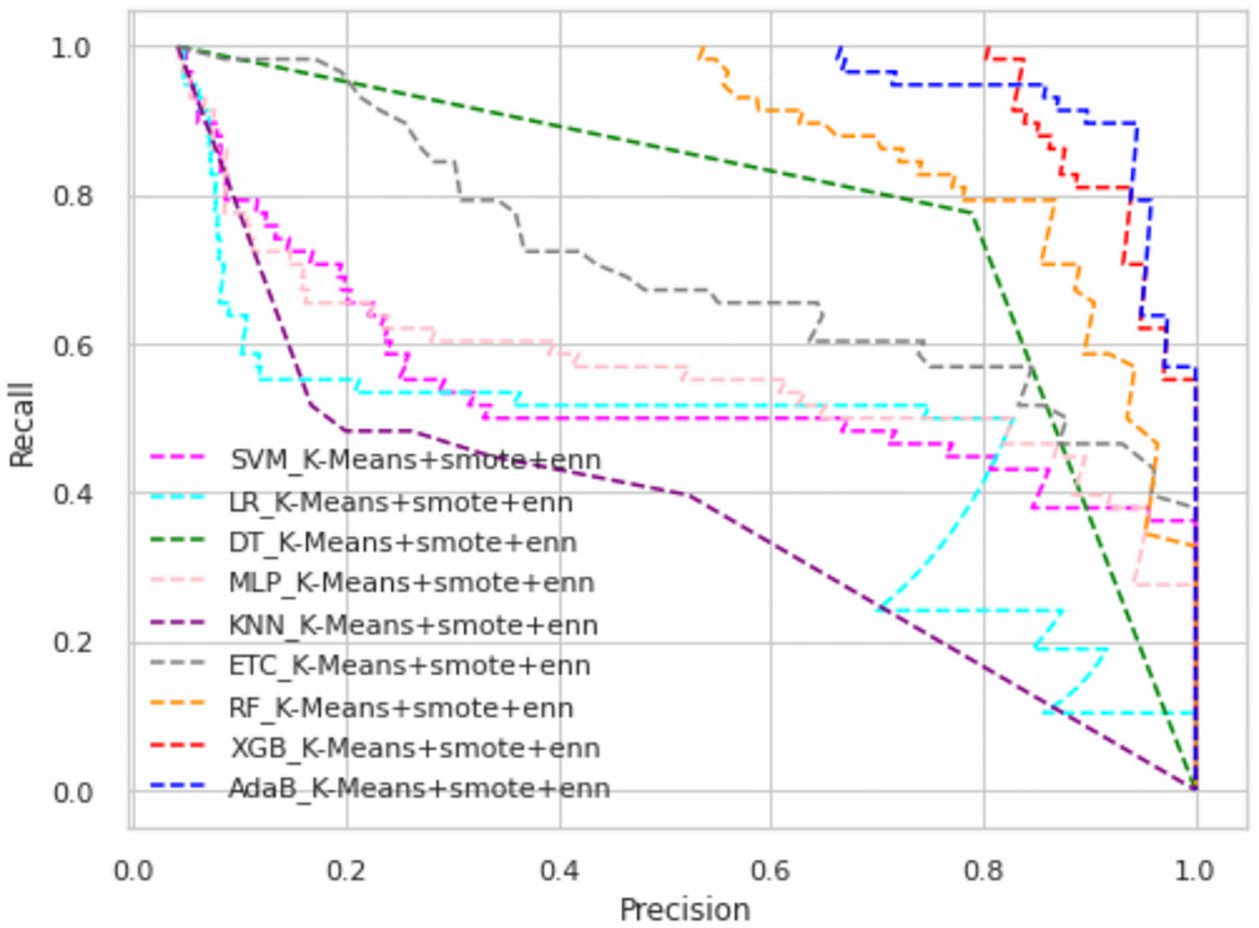
Precision-recall curve of K-means+SMOTE+ENN.

**Fig 6:**
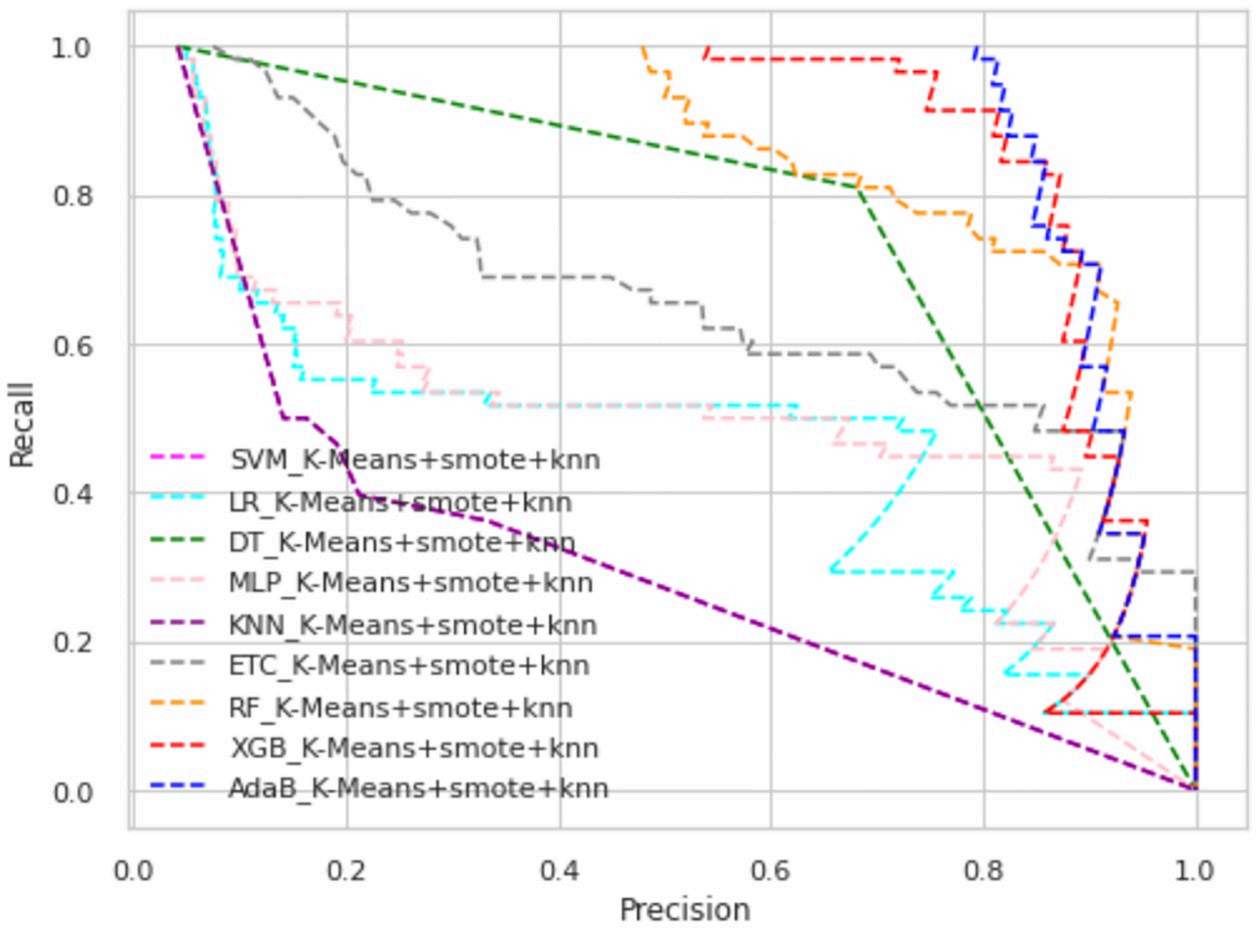
Precision-recall curve of K-means+SMOTE+KNN.

Among all types of classifiers, tree-based classifier performance is higher than statistical models as well as neural network models. Overall, the XGB classifier has shown the best performance in all scenarios. In K-means+SMOTE+ENN, AdaBoost’s and XGB’s ROC values are almost alike, as shown in Fig 4. Overall, AdaBoost and RF also have high ROC values, whereas KNN is placed in the lowest position in these figures. The precision-recall curve provides a visualization of the trade-off between precision and recall at different classification thresholds. In Figs 5 and 6, we have represented the precision-recall curve of our proposed schemes, K-means+SMOTE+ENN and K-means+SMOTE+KNN, respectively.

### Performance of our proposed balancing technique on an independent dataset

Overfitting is a common concern in ML models, where a model performs well on training data but fails to generalize to new, unseen data. In this section, we have evaluated the effectiveness of our proposed technique in mitigating overfitting by employing an independent dataset. We have considered an imbalanced heart disease dataset [48] and used our proposed scheme, K-means+SMOTE+ENN, to balance the dataset. The performance of all the ML methods before and after applying our proposed scheme for the heart disease dataset is presented in Tables 7 and 8. As we can see, the ML models performed well with the balanced data from our proposed balancing technique. This result depicted our model’s ability to mitigate overfitting, offering transparency, robustness, and confidence in its potential for real-world deployment and generalization across diverse datasets.

**Table 7:** The performance of ML classifiers on the Hungarian heart disease dataset without balancing.

ML Models	Accuracy	Precision	Recall	F1 score
AdaBoost	0.8901	0.8856	0.8831	0.8841
DT	0.8812	0.8802	0.8892	0.8858
ETC	0.8578	0.8417	0.8567	0.8509
KNN	0.6652	0.6574	0.6653	0.6566
LR	0.8369	0.8467	0.8303	0.8331
MLP	0.8359	0.8393	0.8364	0.8378
RF	0.9018	0.9087	0.9032	0.9056
SVM	0.6463	0.4151	0.6469	0.5063
XGB	0.9229	0.9178	0.9288	0.9182

**Table 8:** The performance of ML classifiers utilizing K-means+SMOTE+ENN on the Hungarian heart disease dataset.

ML Models	Accuracy	Precision	Recall	F1 score
AdaBoost	0.9012	0.9056	0.9023	0.9039
DT	0.8670	0.8644	0.8605	0.8623
ETC	0.8988	0.8909	0.8972	0.8936
KNN	0.8907	0.8944	0.8902	0.8919
LR	0.8810	0.8812	0.8802	0.8807
MLP	0.8821	0.8841	0.8812	0.8817
RF	0.9289	0.9398	0.9378	0.9388
SVM	0.9212	0.9211	0.9244	0.9226
XGB	0.9291	0.9378	0.9269	0.9274

### Performance of ML classifiers with existing data balancing techniques

To find out the strength of our proposed scheme, we have to compare it with the existing techniques. As we proposed two data balancing techniques, we compared our scheme with the existing four data balancing techniques.

At first, we experimented with an oversampling technique. We chose SMOTE as an oversampling technique to balance the training data. Then, we applied a range of ML models and measured the performance with some performance measure metrics accuracy, precision, recall, F1 score, and AUC value. All nine benchmark ML algorithms’ performance is shown in Table 9 after applying SMOTE. Among all ML algorithms, XGB shows the best performance with SMOTE. On the other hand, KNN and LR have shown the lowest accuracy.

**Table 9:** Performance measure after applying SMOTE.

ML Models	Accuracy	Precision	Recall	F1 score	AUC
AdaBoost	0.9703	0.9826	0.9703	0.9740	0.9962
DT	0.9680	0.9746	0.9609	0.9656	0.9797
ETC	0.9638	0.9632	0.9638	0.9635	0.9015
KNN	0.8699	0.9454	0.8699	0.9009	0.7631
LR	0.8606	0.9445	0.8706	0.9012	0.8273
MLP	0.9017	0.9555	0.9017	0.9227	0.9050
RF	0.9768	0.9761	0.9768	0.9764	0.9903
SVM	0.9140	0.9517	0.9140	0.9294	0.8360
XGB	0.9817	0.9905	0.9877	0.9884	0.9980

After that, we chose NearMiss as an undersampling technique to measure the difference from SMOTE. The performance of all ML classifiers after applying NearMiss is shown in Table 10.

**Table 10:** Performance measure after applying NearMiss.

ML Models	Accuracy	Precision	Recall	F1 score	AUC
Ada	0.9754	0.9845	0.9754	0.9780	0.9926
DT	0.9776	0.9854	0.9776	0.9798	0.9883
ETC	0.3078	0.9476	0.3078	0.4236	0.7665
KNN	0.4703	0.9284	0.4703	0.6038	0.6067
LR	0.5830	0.9401	0.5830	0.7011	0.7475
MLP	0.3807	0.9426	0.3807	0.5095	0.6437
RF	0.9082	0.9712	0.9082	0.9299	0.9892
SVM	0.3460	0.9221	0.3460	0.4751	0.6055
XGB	0.9754	0.9845	0.9754	0.9780	0.9906

Most of the ML models performed worst with NearMiss, as undersampling techniques discard a huge amount of original data. This is the limitation of undersampling techniques. Though SMOTE performed better than NearMiss, the problem with SMOTE is that it creates noises in the dataset. To reduce the noise of this oversampled data, we chose two undersampling techniques: Edited Nearest Neighbour(ENN) and K Nearest Neighbour(KNN). In Table 11, the performance of all ML classifiers is shown after applying SMOTE+ENN to balance the training data. We found that the hybrid data balancing technique performed far better than SMOTE most of the time. Among nine ML models, only the performance of SVM is decreased.

**Table 11:** Performance measure after applying SMOTE+ENN.

ML Models	Accuracy	Precision	Recall	F1 score	AUC
AdaBoost	0.9797	0.9857	0.9797	0.9815	0.9967
DT	0.9768	0.9810	0.9768	0.9783	0.9302
ETC	0.9674	0.9657	0.9674	0.9664	0.9408
KNN	0.8778	0.9424	0.8778	0.9050	0.7190
LR	0.8699	0.9445	0.8699	0.9007	0.8099
MLP	0.9161	0.9520	0.9161	0.9308	0.8450
RF	0.9761	0.9801	0.9761	0.9776	0.9920
SVM	0.8894	0.9470	0.8894	0.9132	0.8422
XGB	0.9826	0.9852	0.9826	0.9835	0.9957

Again, we applied another hybrid technique, SMOTE+KNN, to balance the imbalanced dataset and measure the performance. After applying this balancing technique, ML classifiers’ performance exceeded the previous three existing data balancing techniques, as shown in Table 12. Among all four existing techniques discussed here, SMOTE+KNN has given more accuracy than others, with an accuracy of 98.91% for the XGB classifier.

**Table 12:** Performance measure after applying SMOTE+KNN.

ML Models	Accuracy	Precision	Recall	F1 score	AUC
AdaBoost	0.9848	0.9883	0.9848	0.9858	0.9975
DT	0.9848	0.9855	0.9848	0.9851	0.9261
ETC	0.9696	0.9673	0.9696	0.9682	0.9465
KNN	0.8880	0.9424	0.8880	0.9111	0.7141
LR	0.8612	0.9438	0.8612	0.8953	0.8153
MLP	0.9241	0.9541	0.9241	0.9363	0.8540
RF	0.9797	0.9821	0.9797	0.9806	0.9929
SVM	0.9096	0.9493	0.9096	0.9261	0.8419
XGB	0.9891	0.9906	0.9891	0.9896	0.9971

To observe existing data balancing techniques with our proposed scheme, we can see that accuracy is increased, and other performance measure metrics, i.e., precision, recall, F1-score, and AUC values, are increased for all of the classifiers. This indicated that our proposed scheme classified thyroid disease more accurately, as only accuracy is not able to give a clear picture of any model’s performance.

### Performance comparison

**Fig 7:**
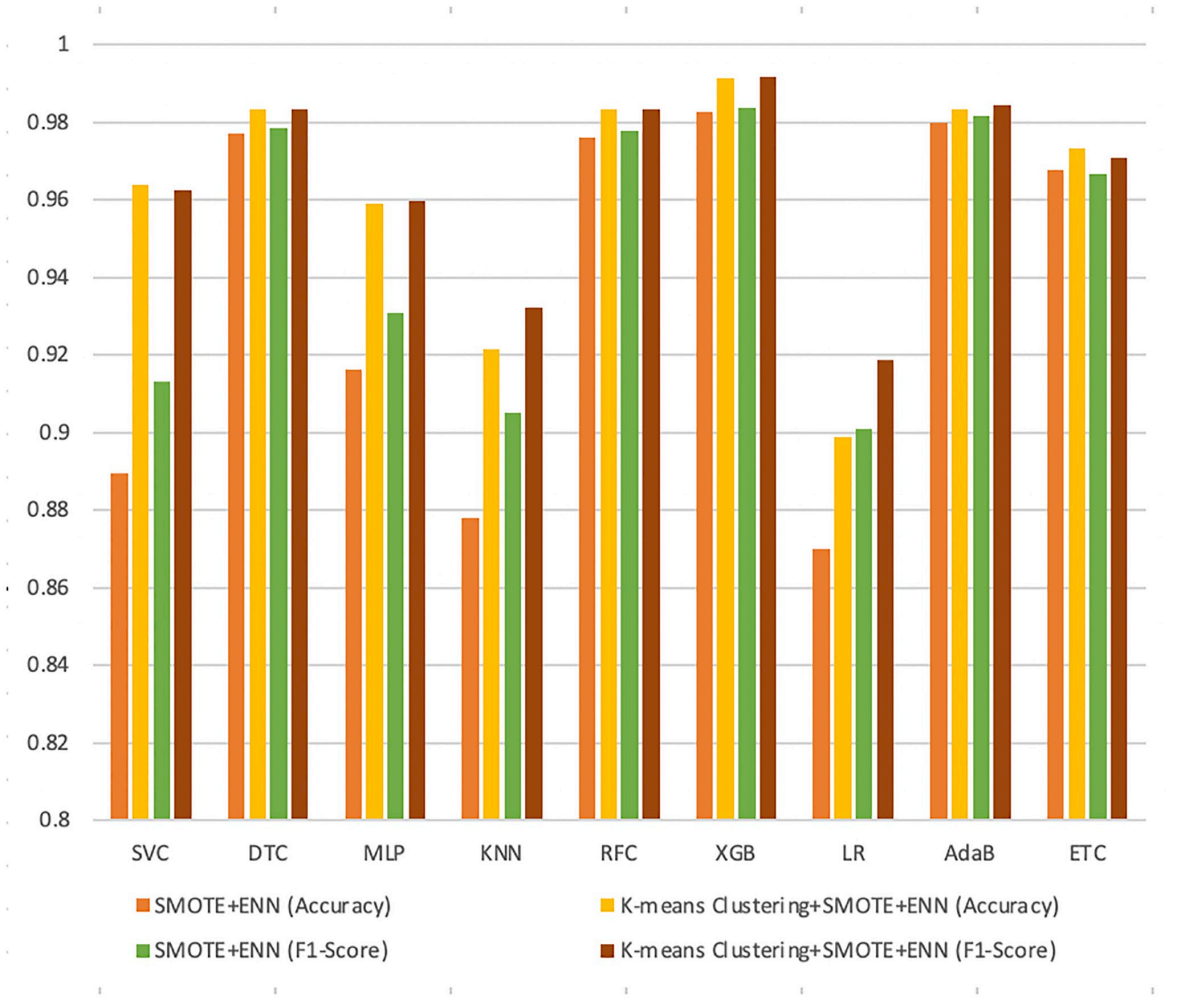
Performance measure between SMOTE+ENN and K-means +SMOTE+ENN.

**Fig 8:**
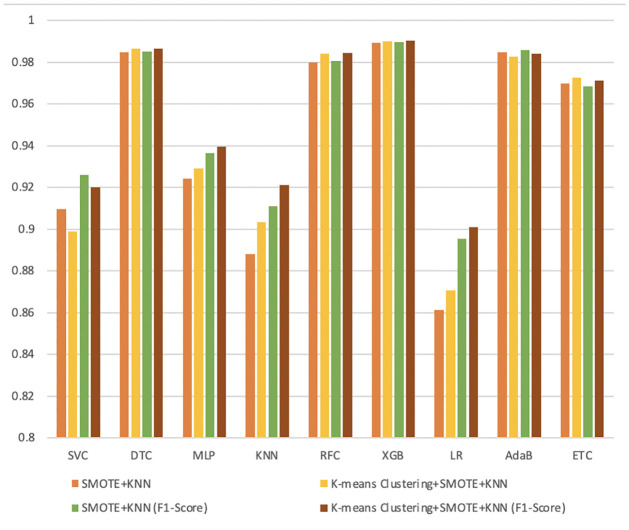
Performance measure between SMOTE+KNN and K-means+SMOTE+KNN.

At the final stage of our analysis, we have summarized our result in Figs 7 and 8. Here, we compared the result of our proposed scheme with existing techniques considering nine ML classifiers.

These two figures illustrate comparing the accuracy and F1-score for nine ML algorithms in our work with existing techniques. It is obvious by comparing the results of a range of ML algorithms that XGB classifier models outperformed other algorithms in these imbalanced datasets. Though the performance of our two proposed data balancing techniques, K-means+SMOTE+ENN and K-means+SMOTE+KNN, outperformed the traditional resampling techniques but between them, K-means+SMOTE+ENN performs better than K-means+SMOTE+KNN.

After taking into account all of the analysis, we can conclude that the hybrid data balancing technique performs much better when we combine it with the clustering process. Hence, we can find the superiority of our proposed data balancing techniques. The Python code of this work is available in the GitHub repository [49].

Among two proposed data balancing techniques, K-means+SMOTE+ENN outperforms K-means+SMOTE+KNN. When using K-means clustering, it significantly alters data distribution within clusters, impacting oversampling techniques. In SMOTE+KNN, reliant on the K-nearest neighbors algorithm, “nearest neighbors” may change due to modified data distribution from K-means, making it less effective for synthetic sample generation. In contrast, SMOTE+ENN is less sensitive to altered cluster distribution, focusing on removing noisy samples. ENN helps make clusters more homogeneous, potentially improving synthetic sample quality. K-means clustering enhances cluster separation, reducing overlapping data points. SMOTE+ENN capitalizes on this separation, while SMOTE+KNN may struggle with tightly clustered neighbors. After employing K-means clustering, SMOTE+ENN benefits from improved separation, enhancing data quality and performance compared to SMOTE+KNN.

### Explainability of thyroid disease prediction

The system remains uninterpretable since it cannot explain the function of each feature predicting thyroid disease. The proposed model is enhanced with an explainable AI (XAI) to explore its interpretability, which allows human users to understand and believe the output and outcomes produced by the black box ML algorithms. To identify the local and global interpretability of our proposed model, we used XAI tools such as SHAP and SHAPASH. These tools helped us to find out the top features, how they are associated with thyroid disease and the individual effect of the features on the performance of the classifiers.

#### Global explainability

**Fig 9:**
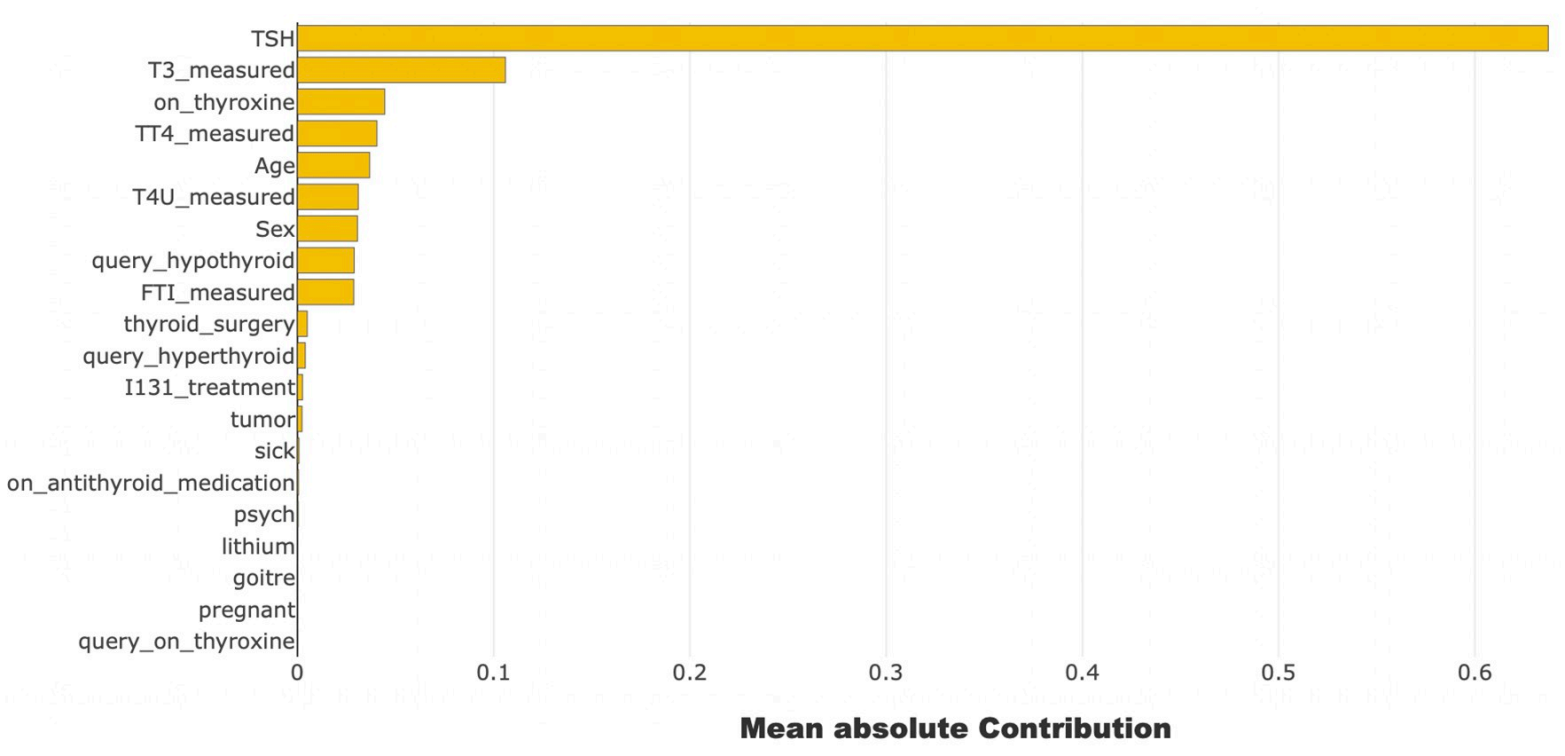
Feature importance of thyroid disease dataset using Shapash.

We applied XAI tools on the XGBoost model, which performed best in classifying thyroid disease. Among all of the features of thyroid disease, we can see that *TSH* is the most important feature to identify thyroid disease from Fig 9. The contribution level of each feature is shown in this figure by using Shapash.

**Fig 10:**
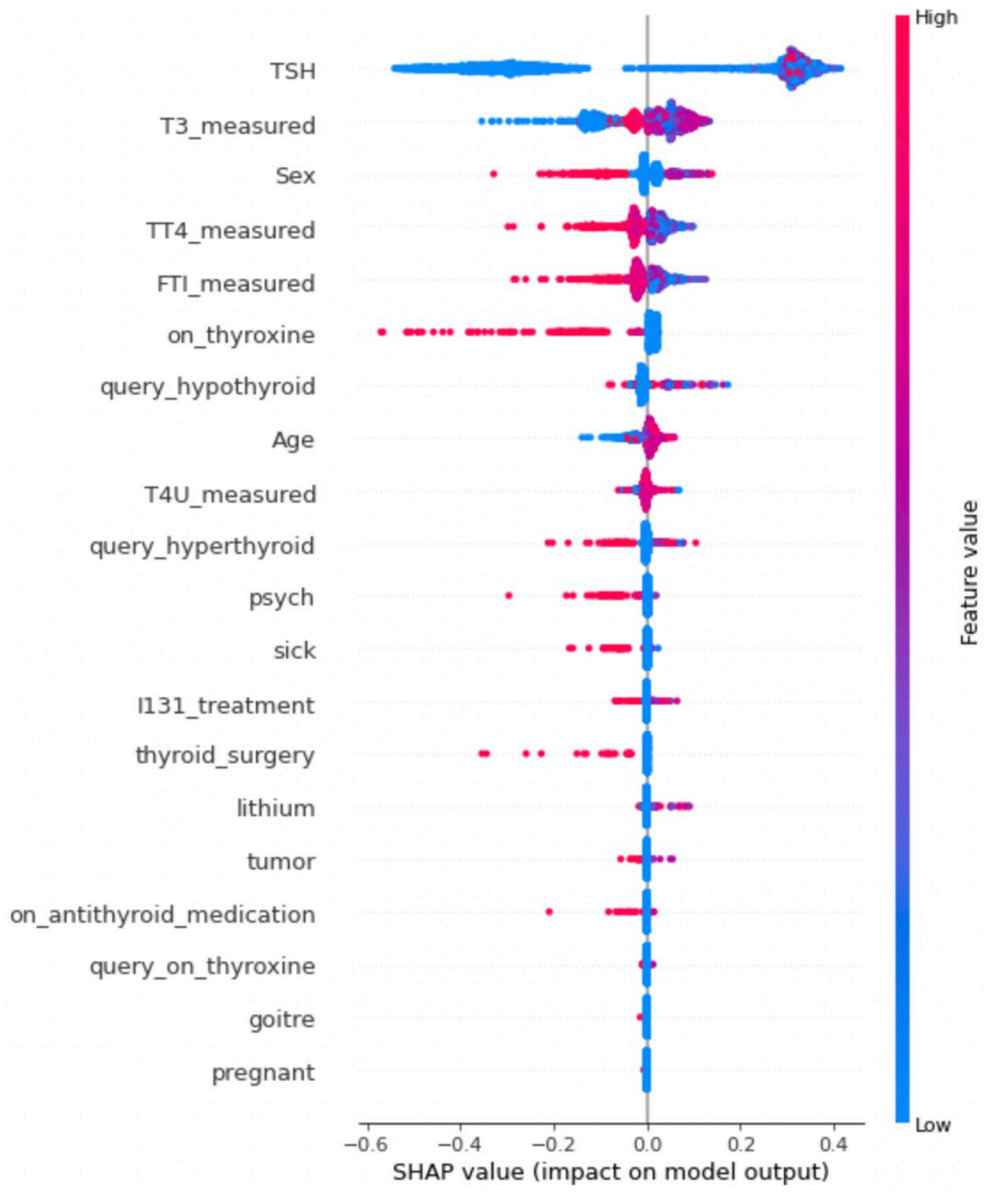
Impact of the features on model output using SHAP value.

Another plot is shown in Fig 10 by using SHAP to explain the significance of each feature on a particular sample in a sorted way. The top features have a higher feature relevance since they contribute more to the model than the bottom ones. This plot tells us that the features of *Sex*, *TT4_measured*, *FTI_measured*, *on_thyroxine* have a negative impact on the model for their high value. Similarly, for the low value of *TT4_measured*, *FTI_measured*, *on_thyroxine* has positive impact on model.

#### Local explainability

**Fig 11:**
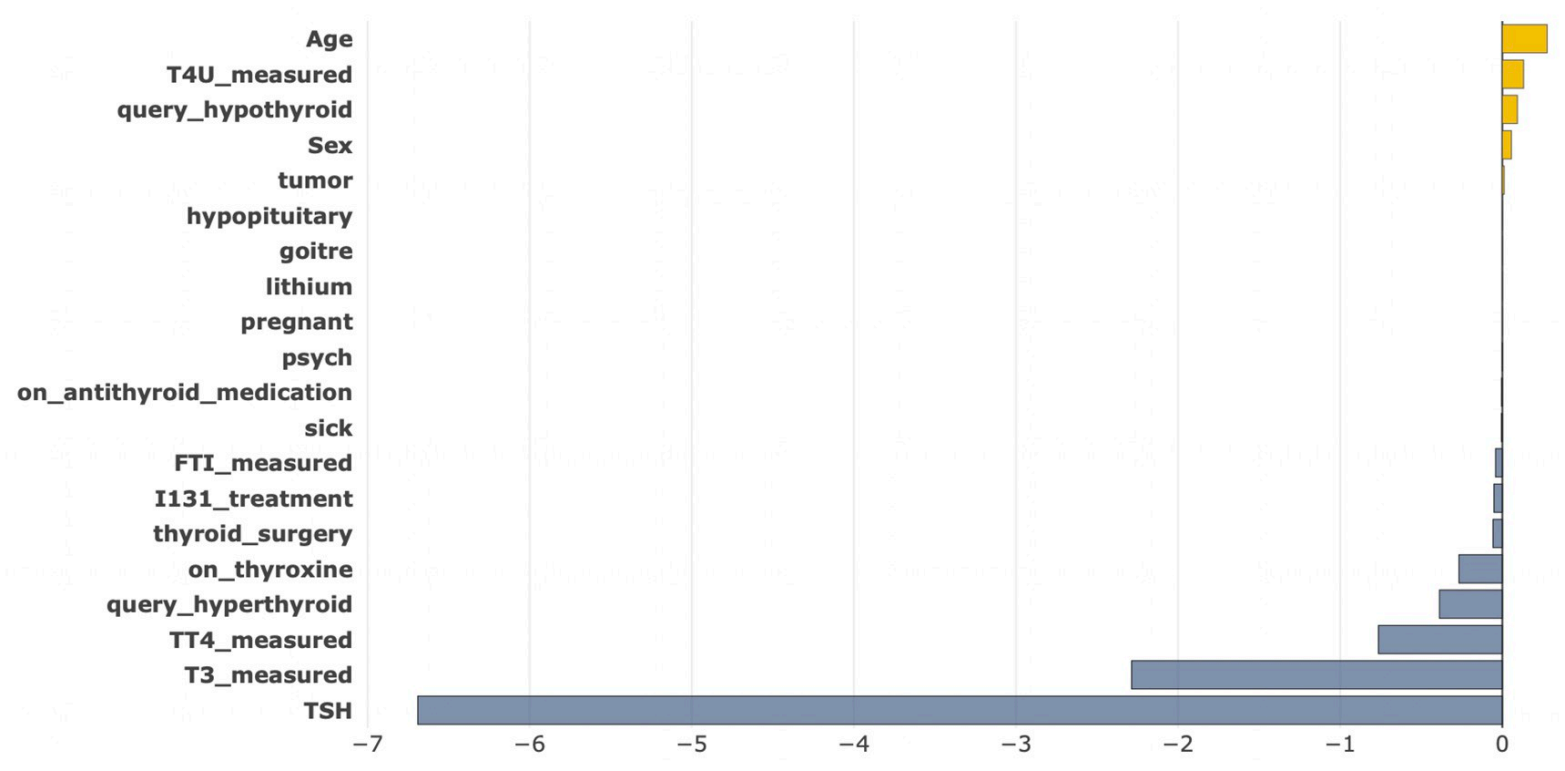
Local explanation for not having a thyroid disease for a random instance.

A global explanation is not enough to know more precisely about the impact of each feature that affected any particular patient. That’s why we explained our model locally using Shapash and SHAP to make our results more trustworthy and transparent for medical professionals. For an individual patient, when predicting not having thyroid disease in Fig 11, we can see that the factors *Age*, *T4U_measured*, *query_hypothyroid* and *Sex* contributed positively. On the other hand, *TSH*, *T3_measured*, *TT4_measured*, *query_hyperthyroid*, *on_thyroxine*, *thyroid_surgery*, *FTI_measured*, and *I131_treatment* contributed negatively and have a high importance on having thyroid disease. As a result, our model predicted a low chance of having thyroid disease.

**Fig 12:**
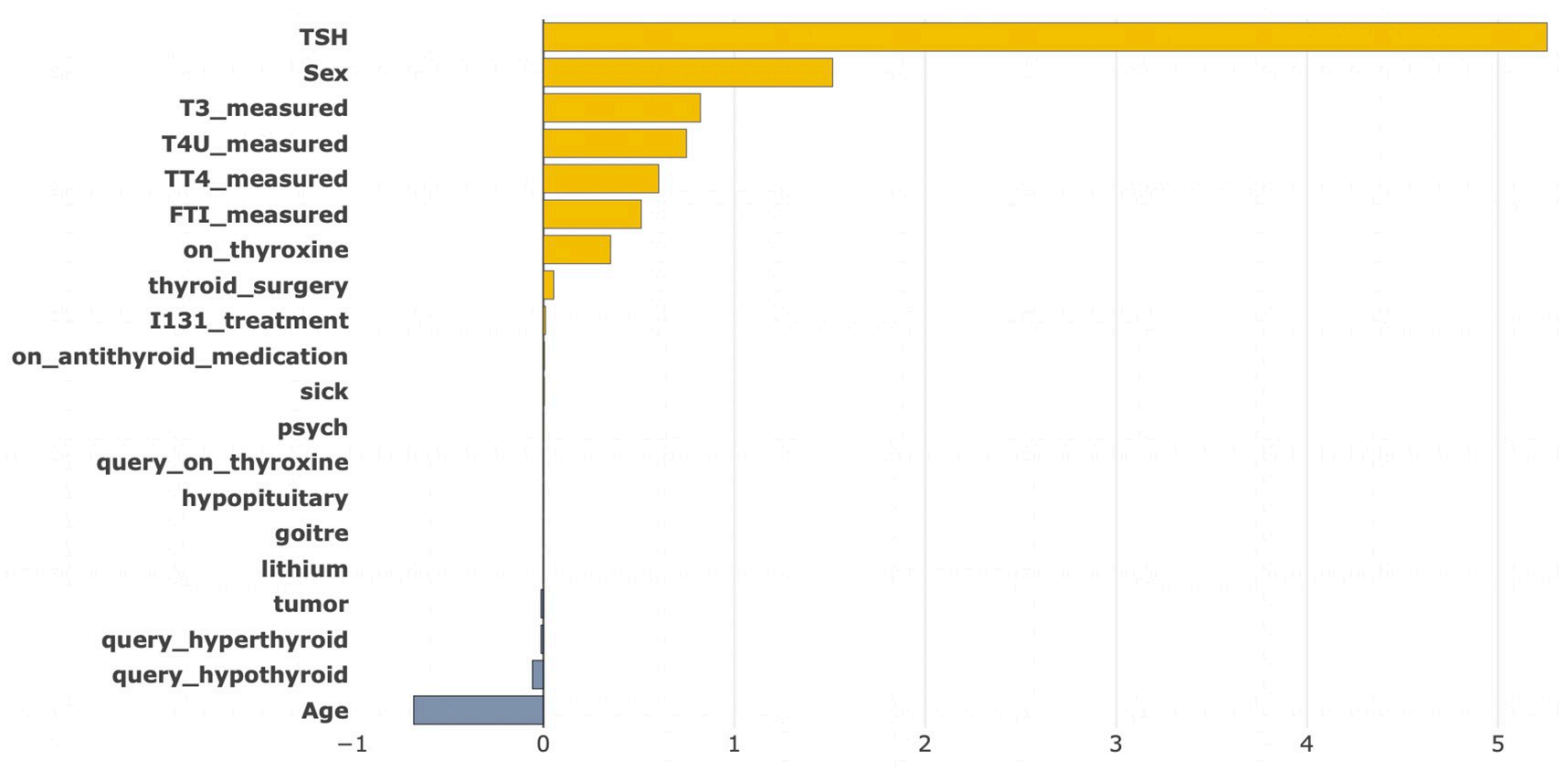
Local explanations for having a thyroid disease for a random instance.

Fig 12 shows local explainability for having thyroid disease using Shapash. We can see that *TSH*, *T3_measured*, *TT4_measured*, *T4U_measured*, *FTI_measured*, *Sex* contributed positively which are the top features for having a thyroid disease for any patient which we have shown in global explanation. Only *Age* and *query_hypothyroid* have some negative impact in this case. For this reason, our model has predicted that this patient has a high chance of having thyroid disease.

**Fig 13:**
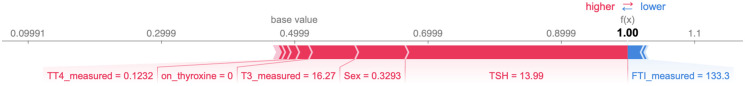
Local explanations for having a thyroid disease for a random instance.

Using SHAP, a visualization is shown in Fig 13 for a patient diagnosing thyroid disease. The major features value for having thyroid disease are *TSH* = 13.99, *Sex* = 0.3293, *T3_measured* = 16.27, *on_thyroxine* = 0, *TT4_measured* = 0.1232. For these feature values, our model predicts this patient has thyroid disease.

**Fig 14:**
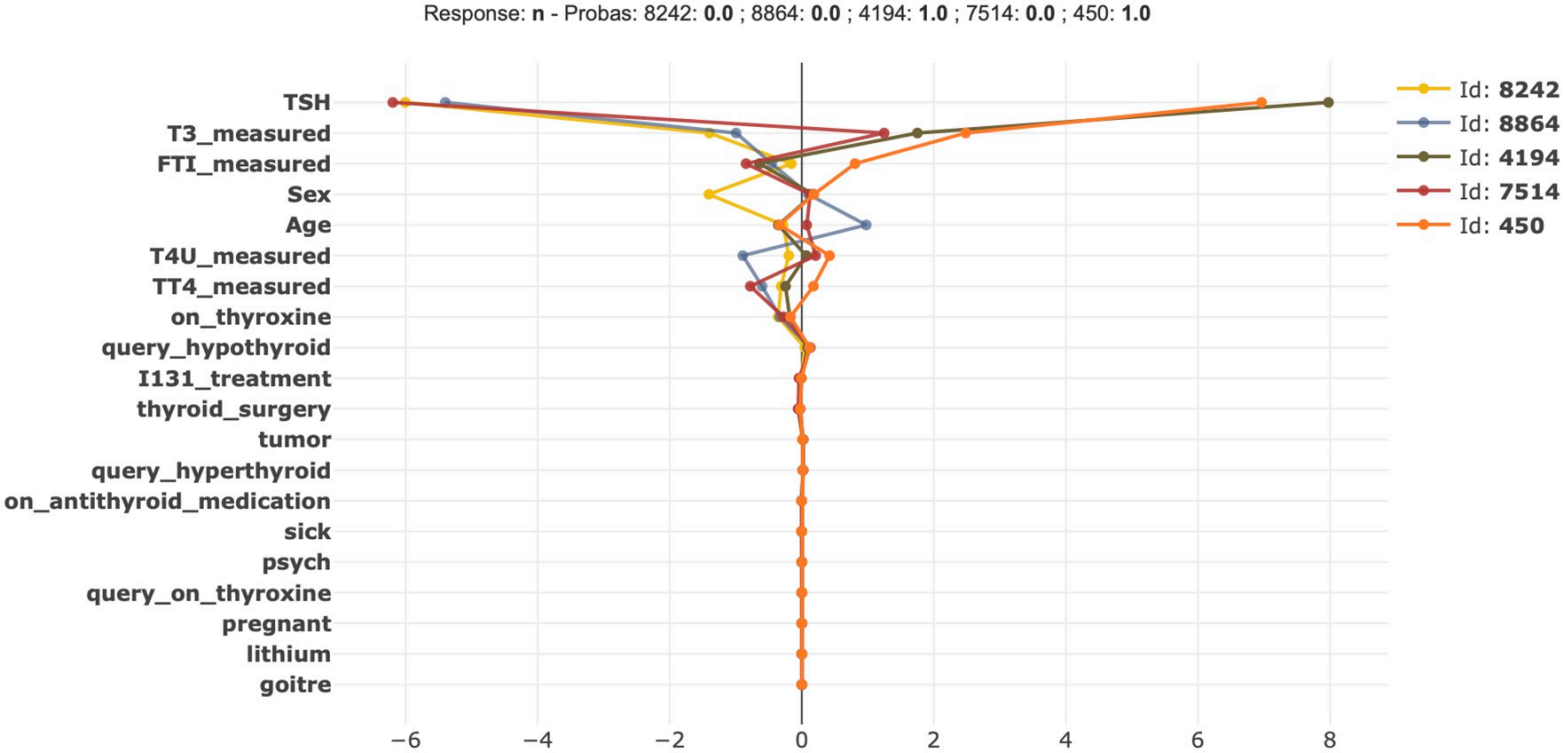
Comparison plot shows the effect of some instances on a model for having thyroid disease.

In Fig 14, a comparison plot shows the effects of the features for four patients diagnosing thyroid disease using Shapash. For ID:4194 and ID:450, the important factors like *TSH*, *T3_measured*, *FTI_measured* positive value significantly influenced our model with a 100% likelihood of getting thyroid disease. For the rest of the three patients, these important features contributed negatively; that’s why our model predicts they have no chance of having thyroid disease.

### Thyroid disease model explainability with ground truth explanation

To explain our black box ML model, we have used XAI tools, which have been explained globally as well as locally. After taking into account these explanations, our models’ working procedure is easily understandable. But one question still arises: “Is the explanation of XAI tools trustworthy?”. To answer this question, we surveyed the domain experts (Doctors and senior medical students). This survey was conducted in two phases. In the first phase, we conducted interviews with doctors, and in the second phase, a mini-survey was conducted online.

#### Structured interviews

Five participants were questioned via structured interviews to get their thoughts on potential indicators of thyroid illness. All the participants were Bangladeshi medical doctors: three of them were female, and two of them were male. According to their opinion, we counted the frequency and ranked the features in Table 13. We got two rank 1 features, nine rank 2 features, and 9 rank 3 features. The rank 1 features are much more important, and we considered them as high-ranked features; the rank 2 features are medium, and the rank 3 features are low-ranked or low-category features.

**Table 13:** Rank and frequency of the domain expert’s opinion.

Feature	D1	D2	D3	D4	D5	Frequency	Rank
*TSH*	1	1	1	1	1	5	1
*T3_measured*	1	1	1	1	1	5	1
*on_thyroxine*	1	1	1	1	0	4	2
*TT4_measured*	1	0	1	0	1	3	2
*Age*	1	0	1	1	1	4	2
*T4U_measured*	1	0	1	1	1	4	2
*Sex*	1	1	1	0	1	4	2
*query_hypothyroid*	0	1	0	1	1	3	2
*FTI_measured*	1	1	1	1	0	4	2
*I131_treatment*	1	1	1	1	0	4	2
*goitre*	1	1	1	1	0	4	2
*thyroid_surgery*	1	0	0	0	0	1	3
*query_hyperthyroid*	1	0	0	1	0	2	3
*tumor*	0	1	0	0	0	1	3
*sick*	0	0	0	0	1	1	3
*on_antithyroid_medication*	1	0	0	0	0	1	3
*psych*	0	1	0	0	0	1	3
*lithium*	0	0	0	1	0	1	3
*pregnant*	1	0	1	0	0	2	3
*query_on_thyroxine*	0	1	0	0	1	2	3

#### Structured survey

In the second stage, we conducted another survey on doctors and medical final-year students. By email, participants were invited to take part in the study. The organized survey was distributed to over 100 potential participants, and 30% of them responded. Google Forms was used to create the questions. Each query is related to one of the stated features. Participants were asked to indicate whether they thought about each particular characteristic while diagnosing or predicting thyroid illness. If so, the priority or importance of this particular attribute is assessed on a scale of one to five, with one denoting negligible importance and five denoting significant significance. From the survey, it is identified that thyroid disease is a major problem worldwide. 100% of the participants agreed with the question, “Do you think Thyroid disease is now a common problem worldwide?”. Among the 30 participants, all believe the hormonal problem is the main cause of thyroid disease. About 70% of them believe the genomics problem is also responsible for this disease, and only 12% of them agree that it can be caused by food habits or lifestyles.

**Fig 15:**
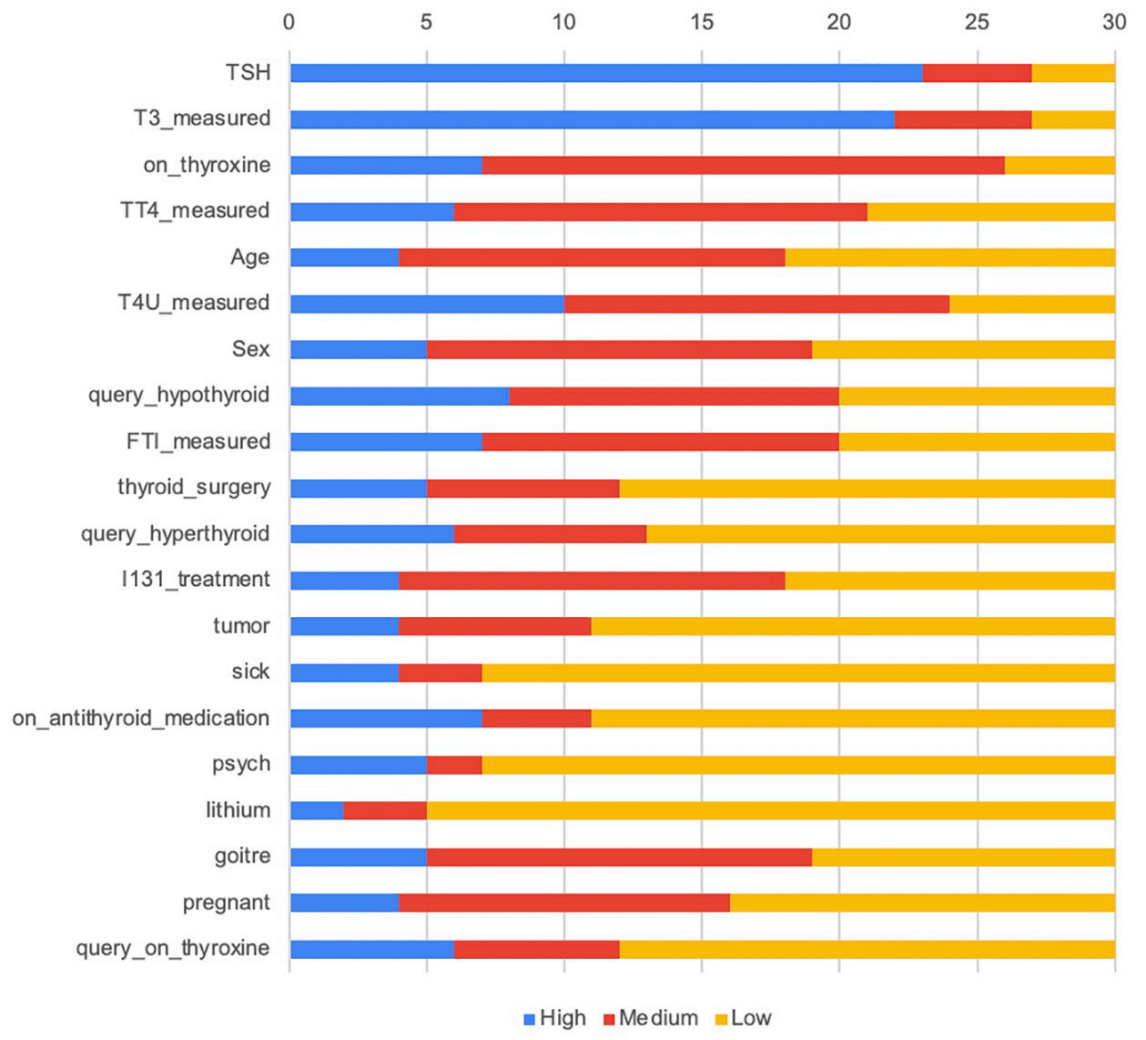
Plot of the summarized responses from survey.

In Table 14, we divided our features into three categories. The features that got the value 5 or 4 in the survey are considered high-ranked features; those that got 3 are in the medium category, and the rest of the features are in the low category or low-ranked features. We have plotted these features in Fig 15.

**Table 14:** The summarized responses from the survey in three categories.

Feature	High	Medium	Low
*TSH*	23	4	3
*T3_measured*	22	5	3
*on_thyroxine*	7	19	4
*TT4_measured*	6	15	9
*Age*	4	14	12
*T4U_measured*	10	14	6
*Sex*	5	14	11
*query_hypothyroid*	8	12	10
*FTI_measured*	7	13	10
*thyroid_surgery*	5	7	18
*query_hyperthyroid*	6	7	17
*I131_treatment*	4	14	12
*tumor*	4	7	19
*sick*	4	3	23
*on_antithyroid_medication*	7	4	19
*psych*	5	2	23
*lithium*	2	3	25
*goitre*	5	14	11
*pregnant*	4	13	13
*query_on_thyroxine*	6	6	18

**Fig 16:**
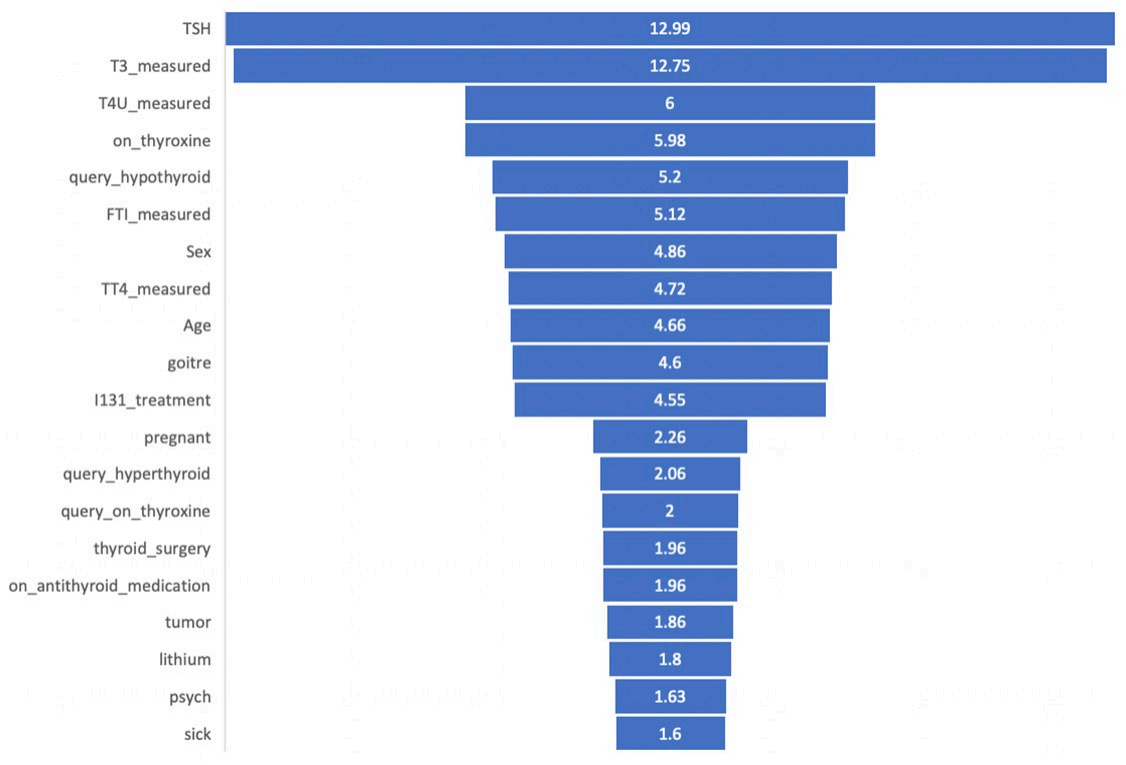
Rank and achieve the score of the features from the survey.

We gave weight 3 to the high-ranked features, weight 2 for the medium-ranked features, and weight 1 to the low-rank features. After adding weight to the survey values, we get the final output that is meaningful to the output of XAI tools. The features are shown in decreasing order in Fig 16.

#### Mapping expert knowledge to XAI tools

To predict thyroid disease, there are 22 features in our dataset. We considered 20 features as one is the target column and a null column. From these features, we got a ranking list according to the features’ importance of having thyroid disease using XAI tools, which were applied in our model. After completing the survey, we have another ranking list of all the features of our dataset, where ranking is made according to the domain expert. Finally, we mapped these two lists to see the difference between AI and ground truth.

The top features are in the same order and the same group in both cases, as shown in Table 15. But in medium-ranked and low-ranked features, rankings are different. According to XAI tools, *goitre* and *I131_treatment* is low importance levels, whereas they seem to have medium importance levels, according to domain experts. Again, *on_thyroxin* feature is the top among medium-ranked features, but it gets 2nd position but is still in the medium category.

**Table 15:** Features ranking according to XAI tools and domain experts.

Feature Name	XAI Level	Expert Level	XAI Serial	Expert Serial
*TSH*	High	High	1	1
*T3_measured*	High	High	2	2
*on_thyroxine*	Medium	Medium	3	4
*TT4_measured*	Medium	Medium	4	8
*Age*	Medium	Medium	5	9
*T4U_measured*	Medium	Medium	6	3
*Sex*	Medium	Medium	7	7
*qyery_hypothyroid*	Medium	Medium	8	5
*FTI_measured*	Medium	Medium	9	6
*thyroid_surgery*	Low	Low	10	15
*qyery_hyperthyroid*	Low	Low	11	13
*I131_treatment*	Low	Medium	12	11
*tumor*	Low	Low	13	17
*sick*	Low	Low	14	20
*on_antithyroid_medication*	Low	Low	15	16
*psych*	Low	Low	16	19
*lithium*	Low	Low	17	18
*goitre*	Low	Medium	18	10
*pregnant*	Low	Low	19	12
*query_on_thyroxine*	Low	Low	20	14

From the explanations of XAI tools and medical experts, we observed that most of the features are in the same categories, but the orders differ. It can be a lack of XAI tools’ understanding or the data differences between the foreign countries and Bangladeshi surveys. Mapping expert knowledge to eXplainable Artificial Intelligence (XAI) tools involves integrating domain-specific insights, expertise, and guidelines into machine learning models to enhance their transparency and interpretability. XAI tools aim to make complex AI models understandable and actionable for humans, particularly experts in various fields.

### Comparison with existing state-of-art methods

In Table 16, we have comprehensively compared our proposed scheme and state-of-the-art methods, evaluating various dimensions to uncover strengths and weaknesses.

**Table 16:** Comparison table between our proposed scheme and the existing state-of-the-art methods.

Ref.	Year	Dataset Details	Preprocessing Steps	Models	Best Performance
[4]	2023	2800 instances, 28 attributes (UCI)	Resampling: SMOTE Feature Selection(FS): RFE and LASSO	SVM, AB, DT, GB, KNN, RF.	RF+LASSO 99% accuracy
[5]	2022	9172 instances, 31attributes (UCI)	FS: FFS, BFE, BiDFE and MLFS	ML: RF, LR, SVM,AdB,GBM DL: CNN, LSTM, CNN-LSTM	MLFS+RF 99% accuracy
[16]	2022	7200 instances, 25 attributes (UCI)	Data augmentation, import Alex net	DT, RF, KNN, and ANN	RF 94.8% accuracy
[17]	2022	3162 instances, 25 attributes (UCI)	Resampling: SMOTE and RandomUnder-Sampler FS: PCA	ANN,CatBoost, XGBoost, RF, LightGBM,DT, ET,SVM,KNN and GNB	ANN Accuracy 0.9587
[18]	2022	3152 instances, 23 attributes (UCI)	FS: PCA, SVD, DT	KNN (K = 3, 5,7), and NN	NN+DT 98.70%
[19]	2020	i.807 instances (clinical data of Kashmi), ii.215 instances (UCI)	N/A	LR, DT, KNN	KNN 96.875%
[20]	2022	215 instances, 5 attributes (UCI)	Feature selection using XGBoost function	DT,LR,KNN, XGB	XGBoost 98.59%
[21]	2020	7200 instances, 21 attributes (UCI)	FS: PC, SU, one-R classifier and RAE	MMLP	MMLP+Pearson’s correlation 99%
[29]	2022	215 instances,5 attributes(UCI)	N/A	LR	XAI (SHAP)
**Proposed**		6916 instances, 22 attributes (UCI)	Proposed techniques: K-means+SMOTE+ENN & K-means+SMOTE+KNN	SVM,DT,MLP,KNN,RF,XGB, LR, AdB, ETC.	XGB 99.13% XAI (SHAP, Shapash)

The comparison emphasized the significance of our approach. Additionally, we have assessed the interpretability of results from the best-performing ML method within our scheme, providing insights into the clarity of the decision-making process compared to state-of-the-art alternatives. This thorough comparison yields a nuanced understanding of our proposed scheme’s position relative to existing methods, offering valuable insights for researchers and practitioners classifying thyroid disease.

### Discussion

According to the objectives, we compared our proposed data balancing techniques with four existing data balancing techniques where our proposed techniques outperformed all the existing techniques. Among all nine ML classifiers, XGBoost has shown the highest performance with 98.84% F1-score and 99.90% AUC. After that, we interpreted our model to uncover the black box ML model using XAI tools. To justify the explanation given by XAI, we surveyed domain experts. Upon mapping expert knowledge to the XAI tool, we found that most of the features’ importance levels match our model’s importance level with some mismatch. In summary, mapping expert knowledge to XAI tools involves a collaborative effort to infuse domain expertise into machine learning models’ decision-making processes. This collaboration aims to create models that not only provide accurate predictions but also generate explanations that are meaningful, relevant, and aligned with the expertise of human practitioners.

## Conclusion

This study focused on the significance of using machine learning models for decision assistance in the healthcare sector. As the selected thyroid dataset suffers from imbalanced problems that hampered the performance of ML models, we proposed a cluster-wise data-balancing technique to predict thyroid disease. We proposed a preprocessing pipeline that improves the classifier’s performance to predict thyroid disease. We also tested four existing data-balancing techniques and checked the performance of the classifiers with our proposed data-balancing techniques. Using 5-fold cross-validation, we found that the performance of the classifiers is stable, and accuracy fluctuates in a minimum range. The overall result showed that the proposed data balancing techniques outperformed the existing four data balancing techniques. We also predicted thyroid disease using nine benchmark ML classifiers. The performances of the classifiers are at a satisfactory level in most of the cases. Finally, we employed XAI tools (SHAP, Shapash, LIME) to find the top-performing model XGB’s explainability. We got an order of the features and found the contributions of the features to the model’s performance individually. Besides the global explainability, we found out the local explainability of the model. A survey from the experts validated the outcome of the XAI tools. In a future study, we will check the performance of the proposed balancing technique with multi-label datasets. We will use distributed learning techniques to make a global model in healthcare for a country without gathering data in a central server.

## Supporting information

S1 File Consent form. (PDF)

S2 File Data collection form. (HTML)
