# Supplementary material for: Analysis and interpretability of machine learning models to classify thyroid disease
Source: PLoS One. 2024 May 31;19(5):e0300670. doi: 10.1371/journal.pone.0300670 (PMC11142566; doi:10.1371/journal.pone.0300670)
Supplement: S1 File — (PDF) [file pone.0300670.s001.pdf]

Photo Serial:

Consent Form For Data Collection

Your recommendations and opinions will be utilized for research purposes. Please provide ratings for a questionnaire. All of the data given by you will be confidential and used for research purposes only.

Participant Information

Name: ....Khadija Anam........

Designation: ....Doctor........

Age: ....28........

Gender:

- o Male
- ✓ Female

Teams and Conditions

1. The researchers can use our data for their research purposes.
2. They can edit and modify the data only for the purpose of research.
3. We will not claim the data for personal use or use for other purposes.
4. Only this research group will get the copyright for the dataset.
5. Data can be present in any research article and we have no objection to that case.

I confirm that I have read and understood the terms and conditions of the above study. I understand that data collected during this study will be processed in accordance with data protection law. I agree to take part in the above study and I have no objection on it.

ডা. খাদিজা আনাম
এমবিবিএস, পিজিটি (গাইনী এন্ড অবস)
বিএমডিসি রেজিঃ নং-[illegible]

....
Signature of Participant

Date: 18·8·23

....
Signature of Principle Investigator

Sumya Akter
Lecturer
Dept. of Computer Science and Engineering
Hajee Mohammad Danesh Science and Technology University, Dinajpur-5200

Date: 20·08·23

CamScanner
