# Supplementary material for: Analysis and interpretability of machine learning models to classify thyroid disease
Source: PLoS One. 2024 May 31;19(5):e0300670. doi: 10.1371/journal.pone.0300670 (PMC11142566; doi:10.1371/journal.pone.0300670)
Supplement: S2 File — (HTML) [file pone.0300670.s002.html]

Data collection form - Google Forms    

JavaScript isn't enabled in your browser, so this file can't be opened. Enable and reload.

Send

Questions

Responses

Settings

Section 1 of 1

Form title

Thyroid disease Prediction Using Machine Learning











Form description

This survey is for research purpose. We only use the data for analysis to predict thyroid disease and its explainability.















Email\*

Valid email

This form is collecting emails.Change settings

Are you a Medical Student/ Doctor/ Medical Science Knowledgable person

\*

Question

Are you a Medical Student/ Doctor/ Medical Science Knowledgable person











\*

Question Type

Short answer

Paragraph

Multiple choice

Checkboxes

Dropdown

File upload

Linear scale

Multiple choice grid

Checkbox grid

Date

Time

Description















Loading image…

Caption

Yes

No

Other…

Add option

or

add "Other"

…

Answer key

(0 points)

Require a response in each rowRequiredRequired

Loading...

Loading…

Do you think, Thyroid disease is now a common problem in worldwide?

\*

Question

Do you think, Thyroid disease is now a common problem in worldwide?











\*

Question Type

Short answer

Paragraph

Multiple choice

Checkboxes

Dropdown

File upload

Linear scale

Multiple choice grid

Checkbox grid

Date

Time

Description















Loading image…

Caption

Yes

No

Other…

Add option

or

add "Other"

…

Answer key

(0 points)

Require a response in each rowRequiredRequired

Loading...

Loading…

What are the reason of Thyroid disease now a days? You can select more than one option if you want.

\*

Question

What are the reason of Thyroid disease now a days? You can select more than one option if you want.











\*

Question Type

Short answer

Paragraph

Multiple choice

Checkboxes

Dropdown

File upload

Linear scale

Multiple choice grid

Checkbox grid

Date

Time

Description















Loading image…

Caption

Lifestyle

Food Habit

Hormonal Problem

Genomic Problem

Other…

Add option

or

add "Other"

…

Select at least

Select at most

Select exactly

Number

Custom error text

Answer key

(0 points)

Require a response in each rowRequiredRequired

Loading...

Loading…

Give the score of the features from 1 to 5 as a cause and syndrome of Thyroid Disease. Here, 5 means it's has a great impact and 1 means has a less impact.

\*

Question

Give the score of the features from 1 to 5 as a cause and syndrome of Thyroid Disease. Here, 5 means it's has a great impact and 1 means has a less impact.











\*

Question Type

Short answer

Paragraph

Multiple choice

Checkboxes

Dropdown

File upload

Linear scale

Multiple choice grid

Checkbox grid

Date

Time

Description















Loading image…

Caption

1

2

3

4

5

TSH

T3\_measured

FTI\_measured

TT4\_measured

T4U\_measured

Age

on\_thyroxine

thyroid\_surgery

I131\_treatment

Sex

query\_hypothyroid

query\_hyperthyroid

tumor

lithium

on\_antithyroid\_medication

sick

psych

query\_on\_thyroxine

goitre

pregnant

hypopituitary

Rows

1.

TSH

2.

T3\_measured

3.

FTI\_measured

4.

TT4\_measured

5.

T4U\_measured

6.

Age

7.

on\_thyroxine

8.

thyroid\_surgery

9.

I131\_treatment

10.

Sex

11.

query\_hypothyroid

12.

query\_hyperthyroid

13.

tumor

14.

lithium

15.

on\_antithyroid\_medication

16.

sick

17.

psych

18.

query\_on\_thyroxine

19.

goitre

20.

pregnant

21.

hypopituitary

1.

Other…

22.

Add row

or

add "Other"

Columns

1

2

3

4

5

Other…

Add column

or

add "Other"

…

Answer key

(0 points)

Require a response in each rowRequiredRequired

Loading...

Loading…

Additional Comments

\*

Question

Additional Comments











\*

Question Type

Short answer

Paragraph

Multiple choice

Checkboxes

Dropdown

File upload

Linear scale

Multiple choice grid

Checkbox grid

Date

Time

Description















Loading image…

Caption

Long answer text

Length

Regular expression

Maximum character count

Minimum character count

Number

Custom error text

Answer key

(0 points)

Require a response in each rowRequiredRequired

Loading...

Loading…

Link to Sheets

The number of responses collected by this form may exceed the limit supported by Sheets. If you're having trouble viewing your responses in Sheets, try downloading a .CSV instead.

Not accepting responses

Accepting responses

Message for respondents

This form is no longer accepting responses

Summary

Question

Individual

Insights

Total points distribution

Loading...

Loading responses…

Are you a Medical Student/ Doctor/ Medical Science Knowledgable person

Copy

No responses yet for this question.

Do you think, Thyroid disease is now a common problem in worldwide?

Copy

No responses yet for this question.

What are the reason of Thyroid disease now a days? You can select more than one option if you want.

Copy

No responses yet for this question.

Give the score of the features from 1 to 5 as a cause and syndrome of Thyroid Disease. Here, 5 means it's has a great impact and 1 means has a less impact.

Copy

No responses yet for this question.

Additional Comments

No responses yet for this question.

Waiting for responses

of

5

of

1

Loading...

Loading response…

Grades from previous versions of this form are hidden. Learn more

Settings

Make this a quiz

Assign point values, set answers, and automatically provide feedback

Release grades

Immediately after each submission

Later, after manual review

Turns on Responses → Collect email addresses

Respondent settings

Missed questions

Respondents can see which questions were answered incorrectly

Correct answers

Respondents can see correct answers after grades are released

Point values

Respondents can see total points and points received for each question

Global quiz defaults

Default question point value

Point values for every new question

points

Responses

Manage how responses are collected and protected

Collect email addresses

Required by Locked mode

Respondents will manually enter their email response

Do not collect

Verified

Responder input

Send responders a copy of their response

Disabled by Locked mode

Requires Collect email addresses

Off

When requested

Always

Allow response editing

Disabled by Locked mode

Responses can be changed after being submitted

Requires sign in

Restrict to users in and its trusted organizations

Required by Locked mode

Respondents will be required to sign in to Google. Learn more

Limit to 1 response

Required by Locked mode

Respondents will be required to sign in to Google.

File upload

Total size limit for all uploaded files

This limit is smaller than the max size of a single response.

Responses will not be accepted after the limit is reached. Learn more

1 GB

10 GB

100 GB

1 TB

Presentation

Manage how the form and responses are presented

Form presentation

Show progress bar

Shuffle question order

After submission

Confirmation message

Your response has been recorded

Edit

Confirmation message

Your response has been recorded

Save

Cancel

Show link to submit another response

Disabled by Locked mode

Disabled by Limit to 1 response

View results summary

Disabled by Locked mode

Share results summary with respondents. Important details

Restrictions:

Disable autosave for all respondents

Respondents will lose their progress if their browser closes or refreshes

Defaults

Form defaults

Settings applied to this form and new forms

Collect email addresses by default

Required by Locked mode

Do not collect

Verified

Responder input

Question defaults

Settings applied to all new questions

Make questions required by default

Google Account

Sumya Akter

sumya.hstu@gmail.com

.
